# Prognostic value of Alzheimer's disease plasma biomarkers in the oldest-old: a prospective primary care-based study

**DOI:** 10.1016/j.lanepe.2024.101030

**Published:** 2024-08-16

**Authors:** Pamela V. Martino-Adami, Madhurima Chatterjee, Luca Kleineidam, Siegfried Weyerer, Horst Bickel, Birgitt Wiese, Steffi G. Riedel-Heller, Martin Scherer, Kaj Blennow, Henrik Zetterberg, Michael Wagner, Anja Schneider, Alfredo Ramirez

**Affiliations:** a Division of Neurogenetics and Molecular Psychiatry, Department of Psychiatry and Psychotherapy, Faculty of Medicine and University Hospital Cologne, University of Cologne, Kerpener Str. 62, 50937, Cologne, Germany; b German Center for Neurodegenerative Diseases (DZNE), Venusberg-Campus 1, 53127, Bonn, Germany; c Department of Old Age Psychiatry and Cognitive Disorders, University Hospital Bonn, Medical Faculty, Venusberg-Campus 1, 53127, Bonn, Germany; d Medical Faculty Mannheim, Heidelberg University, Germany; e Department of Psychiatry, Technical University of Munich, Germany; f Institute of General Practice, Hannover Medical School, Germany; g Institute of Social Medicine, Occupational Health and Public Health, University of Leipzig, Germany; h Department of Primary Medical Care, Center for Psychosocial Medicine, University Medical Center, Hamburg-Eppendorf, Germany; i Department of Psychiatry and Neurochemistry, The Sahlgrenska Academy at the University of Gothenburg, Mölndal, Sweden; j Clinical Neurochemistry Laboratory, Sahlgrenska University Hospital, Gothenburg, Sweden; k Paris Brain Institute, ICM, Pitié-Salpêtrière Hospital, Sorbonne University, Paris, France; l Neurodegenerative Disorder Research Center, Division of Life Sciences and Medicine, and Department of Neurology, Institute on Aging and Brain Disorders, University of Science and Technology of China and First Affiliated Hospital of USTC, Hefei, PR China; m Department of Neurodegenerative Disease, UCL Institute of Neurology, Queen Square, London, UK; n UK Dementia Research Institute at UCL, London, UK; o Hong Kong Center for Neurodegenerative Diseases, Clear Water Bay, Hong Kong, China; p Wisconsin Alzheimer's Disease Research Center, University of Wisconsin School of Medicine and Public Health, University of Wisconsin-Madison, Madison, WI, USA; q Department of Psychiatry and Glenn Biggs Institute for Alzheimer's and Neurodegenerative Diseases, 7703 Floyd Curl Drive, 78229, San Antonio, TX, USA; r Cologne Excellence Cluster on Cellular Stress Responses in Aging-associated Diseases (CECAD), University of Cologne, Joseph-Stelzmann-Straße 26, 50931, Cologne, Germany

**Keywords:** Alzheimer's disease, Plasma biomarkers, Oldest-old population, Primary care, Cognitive impairment, Disease prognosis

## Abstract

**Background:**

Blood-based biomarkers offer a promising, less invasive, and more cost-effective alternative for Alzheimer's disease screening compared to cerebrospinal fluid or imaging biomarkers. However, they have been extensively studied only in memory clinic-based cohorts. We aimed to validate them in a more heterogeneous, older patient population from primary care.

**Methods:**

We measured plasma Aβ42/Aβ40, P-tau181, NfL, and GFAP in 1007 individuals without dementia, aged 79–94 years, from the longitudinal, primary care-based German AgeCoDe study. We assessed the association with cognitive decline, disease progression, and the capacity to predict future dementia of the Alzheimer's type (DAT). We also evaluated biomarker dynamics in 305 individuals with a follow-up sample (∼8 years later).

**Findings:**

Higher levels of P-tau181 (HR = 1.32 [95% CI: 1.17–1.51]), NfL (HR = 1.19 [95% CI: 1.03–1.36]), and GFAP (HR = 1.36 [95% CI: 1.22–1.52]), and a lower Aβ42/Aβ40 ratio (HR = 0.80 [95% CI: 0.68–0.95]) were associated with an increased risk of progressing to clinically-diagnosed DAT. Additionally, higher levels of P-tau181 (β = −0.49 [95% CI: −0.71 to 0.26]), NfL (β = −0.29 [95% CI: −0.52 to 0.06]), and GFAP (β = −0.60 [95% CI: −0.83 to 0.38]) were linked to faster cognitive decline. A two-step DAT prediction strategy combining initial MMSE with biomarkers improved the identification of individuals in the prodromal stage for potential treatment eligibility. Biomarker levels changed over time, with increases in P-tau181 (β = 0.19 [95% CI: 0.14–0.25]), NfL (β = 2.88 [95% CI: 2.18–3.59]), and GFAP (β = 8.23 [95% CI: 6.71–9.75]). NfL (β = 2.47 [95% CI: 1.04–3.89]) and GFAP (β = 4.45 [95% CI: 1.38–7.51]) exhibited a faster increase in individuals progressing to DAT.

**Interpretation:**

Evaluating plasma biomarkers, alongside brief cognitive assessments, might enhance the precision of risk assessment for DAT progression in primary care.

**Funding:**

10.13039/100010146Alzheimer Forschung Initiative, Bundesministerium für Bildung und Forschung.

**Research in context**

**Evidence before this study**

Cerebrospinal fluid and positron emission tomography biomarkers demonstrate excellent diagnostic properties for Alzheimer's disease; however, their invasiveness, cost, limited availability, and occasional contraindications pose significant challenges. In contrast, blood-based biomarkers present a notable advantage. While high-performing assays for plasma Aβ40, Aβ42, P-tau181, NfL, and GFAP effectively detect Alzheimer's disease and neurodegeneration-related pathological changes, their examination has been primarily focused on clinically based cohorts, often characterised by mild comorbidities. The older patient population frequenting primary care is much more heterogeneous, with mixed pathologies and prevalent comorbidities. We searched PubMed for articles published up to July 1, 2024, on blood-based (“blood OR serum OR plasma”) biomarkers (“biomarkers”) for Alzheimer's disease (“Alzheimer”), without language restrictions. We focused on articles involving individuals from population-based studies (“population-based” OR “community-based” OR “primary care-based”). The body of evidence on the performance of Alzheimer's disease plasma biomarkers in population-based studies is growing. However, these studies were conducted in community-based cohorts with individuals without dementia who were younger than 85 years. To our knowledge, none of them have extensively investigated the prognostic value of Aβ42/Aβ40 ratio, P-tau181, NfL, and GFAP in the oldest old primary care patients without dementia.

**Added value of this study**

We assessed the baseline plasma levels of Aβ40, Aβ42, P-tau181, NfL, and GFAP in 1007 oldest-old (aged 79–94 years), primary care individuals without dementia from the German AgeCoDe study. For a subgroup of 305 participants, we conducted follow-up biomarkers measurements. Our findings revealed that all plasma biomarkers were linked to the risk of progressing to clinically-diagnosed dementia of the Alzheimer's type (DAT), whereas only P-tau181, NfL, and GFAP showed associations with cognitive decline. Employing a two-step strategy for predicting clinically-diagnosed DAT within four years, involving an initial MMSE followed by plasma biomarkers, improved the identification of patients in the prodromal stage who would be eligible for disease-modifying Alzheimer's treatment therapy. Notably, in the subset with follow-up samples, biomarker levels demonstrated changes over time, with NfL and GFAP exhibiting a more rapid increase in participants who progressed to clinically-diagnosed DAT.

**Implications of all the available evidence**

The emerging evidence suggests that effective implementation of plasma biomarkers could improve the precision of risk assessment for progression to DAT within the more heterogeneous, older patient population encountered at primary care. Notably, plasma Aβ42/Aβ40 ratio could serve an early-stage indicator, while NfL as a biomarker for later stages. Plasma Aβ42/Aβ40 ratio and GFAP level, in combination with cognitive testing and basic demographics, could be used in a two-step approach to effectively identify patients in the prodromal stage eligible for treatment, facilitating timely referral to specialised memory centres. Plasma levels of NfL and GFAP could be valuable to monitor disease progression.

## Introduction

Alzheimer's disease (AD) biomarkers have led to a fundamental reconsideration of AD pathogenesis by demonstrating that AD pathology exists from a preclinical stage to the progressively symptomatic stages of subjective cognitive decline (SCD), mild cognitive impairment (MCI), and dementia (dementia of the Alzheimer's type, DAT). They have also provided the research field with a diagnosis framework (A/T/N)^1^^,^^2^ that shifted the definition of AD in living people from a syndrome to a biological construct, improving diagnosis sensitivity and specificity. Cerebrospinal fluid (CSF) and positron emission tomography (PET) biomarkers have excellent diagnostic properties, but they are more invasive (CSF), or more expensive and not widely available (PET), and in some cases contraindicated. In this scenario, blood-based biomarkers promise to offer a major benefit.

High-performing assays for plasma amyloid-beta 40 (Aβ40), amyloid-beta 42 (Aβ42), phosphorylated-tau 181 (P-tau181), neurofilament light chain (NfL), and glial fibrillary acidic protein (GFAP) can detect relatively well AD and neurodegeneration-related pathological changes. Plasma Aβ42/Aβ40 ratio, by correcting inter-individual differences in Aβ processing, can detect cerebral Aβ pathology with high accuracy^3^ but exhibits a rather minor fold change (∼10–12% reduction) between amyloid positive and negative cases.^4^^,^^5^ P-tau181 can accurately discriminate individuals with AD neuropathologic changes from those without, including those with non-AD tau pathology.^6^ NfL and GFAP reflect axonal damage and reactive astrocytes, respectively, and although non-AD specific, they are good indicators for disease progression.^7^^,^^8^

Even though patients with early cognitive symptoms are primarily treated in primary care, memory impairment may not be apparent during a routine general practitioner (GP) office visit unless directly assessed. The lack of accessible and accurate tools to diagnose cognitive impairment in primary care results in a substantial number of diagnoses that are missed or delayed,^9^ leading to lost opportunities for treatment and increasing patient and caregiver burden. Unfortunately, plasma biomarkers have been mainly studied in patients from specialised memory clinics, raising the question of whether these results from memory clinic-based studies can be directly translated to the general population in primary care. The older patient population with cognitive symptoms in primary care is much more heterogeneous, with mixed pathologies and more frequent comorbidities, such as hypercholesterolemia, diabetes, and kidney and cardiovascular diseases. Therefore, answering this question will require validating plasma biomarkers in large, heterogeneous primary care-based studies.

Consequently, we measured the plasma levels of Aβ40, Aβ42, P-tau181, NfL, and GFAP in 1007 participants without dementia, aged 79–94 years and enrolled in the longitudinal population-based German Study on Ageing, Cognition and Dementia in Primary Care Patients (AgeCoDe). We sought to determine the association between plasma biomarker levels and cognitive decline and disease progression, and their capacity to improve the prediction of future clinically-diagnosed DAT that can be obtained with easily accessible and cost-effective measures. Furthermore, to investigate age-related changes, we evaluated plasma biomarker dynamics in a subset of 305 participants with a follow-up (∼8 years later) plasma sample available.

## Methods

### Study participants

Participants in the German Study on Ageing, Cognition, and Dementia in Primary Care Patients (AgeCoDe) were enrolled between 2003 and 2004 across six major German cities: Bonn, Düsseldorf, Hamburg, Leipzig, Mannheim, and Munich. These cities represent urban areas with populations ranging from approximately 300,000 (Mannheim) to nearly 1.8 million (Hamburg). Recruitment was conducted by 19–29 general practitioners (GPs) per site, totalling 138 practitioners across all sites.^10^^,^^11^ Inclusion criteria for potentially eligible patients were at least 75 years of age, absence of dementia according to the judgement of the GP, and at least one contact with the GP within the past 12 months. Neuropsychological and clinical assessments for dementia were based on SIDAM and CERAD.^12^^,^^13^ MCI was defined according to one standard deviation deficit in any cognitive domain in the cognitive test battery implemented in the SIDAM. The level of education received by the patients was measured by the highest completed level and categorised as low (primary school level/elementary education), intermediate (intermediate vocational/general qualification), or high (undergraduate or postgraduate studies/tertiary education) using the Comparative Analysis of Social Mobility in Industrial Nations (CASMIN) international educational classification.^14^ A total of 3327 individuals were enrolled in the study, and blood samples were collected during two time periods: between 2007 and 2009 (approximately 5 years after the AgeCoDe start date) and between 2015 and 2016 (approximately 13 years after the start date). The study subset analysed in this research comprised 1007 individuals without dementia aged 79–94 years during the 2007–2009 period with available plasma samples (considered as the analysis baseline of our study). A subset of 305 participants had matching samples drawn between 2015 and 2016 (approximately 8 years later, follow-up (FU) sample). Detailed information regarding the study design, recruitment process, and diagnostic procedures can be found in the Supplementary Material.

### APOE genotyping

For DNA analysis, leukocyte DNA was isolated with Qiagen blood isolation kit according to the manufacturer's instructions (Qiagen, Germany). *APOE* was genotyped as described before.^15^

### Biomarker measurement

Blood was collected by participants’ GPs in tubes with EDTA, and transported the same day by post at ambient temperature and protected from light. After arrival at the central biorepository in Bonn, samples were centrifuged and plasma was separated, aliquoted, and stored at −80 °C until further use. Plasma levels of Aβ40, Aβ42, NfL, and GFAP were measured using the Neurology 4-plex E Simoa assay kit, and plasma level of P-tau181 using the pTau181 Advantage V2 Simoa assay kit (Quanterix, Billerica, MA). Calibrators were run in duplicates and obvious outlier replicates were masked before curve fitting. Samples were diluted 4-fold and run in singlicates, and results were compensated for the dilution. Two quality control levels were run in duplicates in the beginning and the end of each run. Assays were run using a Simoa HD-X platform at the University of Gothenburg. Since chronic kidney disease has been associated with higher levels of Aβ40, Aβ42, P-tau181, NfL, and total tau,^16^^,^^17^ eGFR was calculated using the CKD-EPI Creatinine–Cystatin Equation (2021)^18^ to include it as a covariate in biomarkers analyses.

### Association between baseline plasma biomarkers and cognitive decline

The effect of baseline Aβ42/Aβ40 ratio or baseline levels of P-tau181, NfL, and GFAP on cognitive decline was assessed with linear mixed-effects models for repeated measures fit by maximum likelihood using the R package *lme4*. The models included all available cognitive data along seven time-points, including those from participants with incomplete follow-up. Missing cognitive data were assumed to be missing at random. Longitudinal MMSE score was used as the outcome measure and time was used as a continuous variable. The models included the fixed effects of time, baseline biomarker ratio/level, baseline age, sex, *APOE* strata, and education level, and the interaction terms between baseline age, sex, *APOE* strata, and education level with the time term. A random slope of time and intercept were included per each subject. MMSE score was normalised to correct for ceiling effects using the R package *NormPsy*, and all continuous independent variables were *z*-transformed to allow comparison. t-tests were calculated with Satterthwaite's method using the R package *lmerTest*. Biomarkers were split into quantiles with low (below 25%), medium (25–75%), and high (over 75%) ratio/levels only for visualisation purposes.

### Association between baseline plasma biomarkers and risk of progressing to mild cognitive impairment or dementia of the Alzheimer's type

The impact of baseline Aβ42/Aβ40 ratio or baseline levels of P-tau181, NfL, and GFAP on the risk of progressing to MCI (evaluated in participants without cognitive impairment) or clinically-diagnosed DAT (evaluated in all participants or only those without cognitive impairment) was assessed using Cox proportional-hazard regressions (continuous data) adjusted for age, sex, and eGFR using the R package *survival*. In the analysis of progression to DAT, individuals who progressed to other types of dementia were considered non-progressors. To allow comparison, all continuous variables were *z*-transformed.

### Prediction of progression to dementia of the Alzheimer's type

Three multivariate logistic models were fitted to predict progression to clinically-diagnosed DAT within four years either in all participants or those with a MMSE score ≤27. All models had clinically-diagnosed DAT progression status as the outcome measure. The first model was fitted including baseline age, sex, *APOE* strata, and MMSE score as predictors. The second model included baseline age, sex, *APOE* strata, plasma Aβ42/Aβ40 ratio, and plasma levels of P-tau181, NfL, and GFAP as predictors. The last model was fitted using baseline age, sex, *APOE* strata, MMSE score, plasma Aβ42/Aβ40 ratio, and plasma levels of P-tau181, NfL, and GFAP as predictors. The area under the ROC curve (AUC) for each model was calculated using the R package *pROC*. The number of true positives, true negatives, false positives, and false negatives, and positive predictive value ($\frac{True\;positives}{True\;positives\;+\;False\;positives}$, also known as precision), negative predictive value ($\frac{True\;negatives}{True\;negatives\;+\;False\;negatives}$), sensitivity, and specificity were calculated using Youden's Index. To allow comparison, all continuous variables were *z*-transformed.

### Plasma biomarkers dynamics

Trajectories of plasma Aβ42/Aβ40 ratio and plasma levels of P-tau181, NfL, and GFAP were assessed with linear mixed-effects models for repeated measures using the R package *lme4*. The models included all available biomarker data along two time-points in a subset of 305 participants with repeated biomarker measurements (baseline and follow-up ∼8 years later). Longitudinal plasma biomarker ratio/level was used as the outcome measure, and the fixed effects included time, baseline age, sex, *APOE* strata, eGFR, clinically-diagnosed DAT-progression status, and the interaction terms between time and the rest of the covariates. Individuals who progressed to other types of dementia were considered non-progressors. Time was treated as a continuous variable. Since only two time-points were available, a random intercept was included per each subject but it was not possible to add a random slope of time for each participant. All continuous covariates were *z*-transformed to allow comparison. t-tests were calculated with Satterthwaite's method using the R package *lmerTest*.

## Results

### Participant characteristics at baseline

**Fig. 1:**
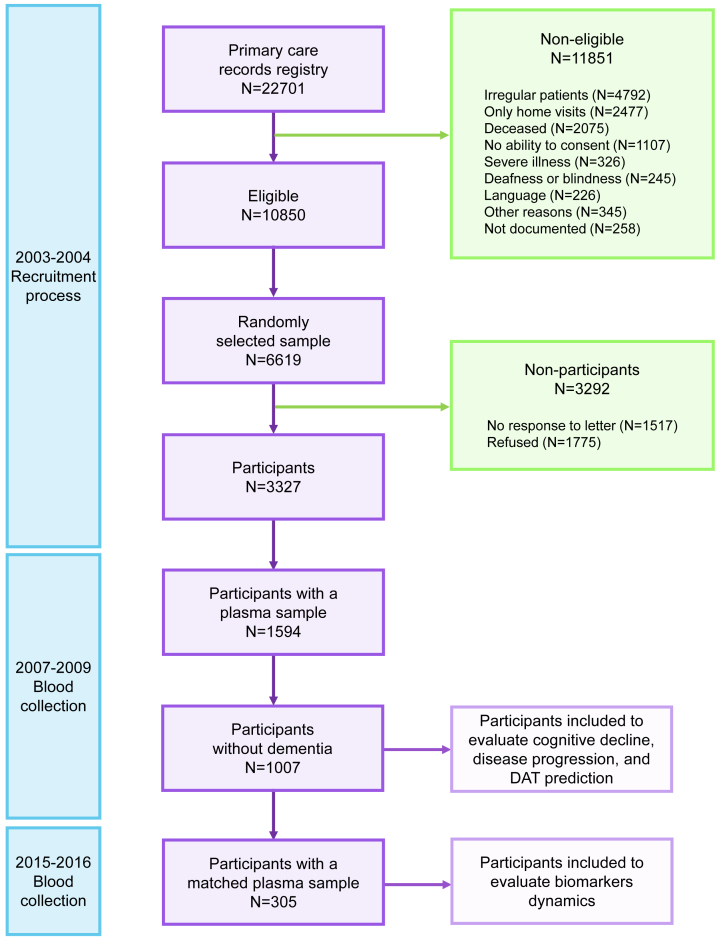
**Flowchart of the selection process of the study participants.** The flowchart indicates the inclusion/exclusion criteria for AgeCoDe recruitment process in 2003–2004 and the selection of participants with plasma samples available for the study analyses. DAT, clinically-diagnosed dementia of the Alzheimer's type.

In this longitudinal study, a total of 1007 participants without dementia were enrolled, with 305 individuals having a follow-up (FU) sample collected approximately 8 years later (Fig. 1). At baseline, the participant demographics indicated that 67.7% were female, and 17.1% possessed at least one copy of an *APOE-ε*4 allele. The median age was 83 years, accompanied by a median MMSE score of 29. Educational backgrounds varied, with 58.1% having elementary education, 29.4% an intermediate level, and 12.5% a high level. Regarding health metrics, the median BMI stood at 25.6, with 15.6% classified as overweight and 45.9% as obese. The median estimated glomerular filtration rate (eGFR) was 58 mL/min/1.73 m^2^, indicating a mild reduction in kidney function likely associated with ageing. Notably, 19.5% exhibited an eGFR lower than 45 mL/min/1.73 m^2^. Additionally, 61.2% of participants were diagnosed with hypercholesterolemia, 23.9% with diabetes, and 25% with heart insufficiency (Table 1).

**Table 1:** Clinical and demographic characteristics of participants without dementia at baseline.

Characteristics	Baseline (N = 1007)
Sex (Female, %)	66.7
*APOE* strata (%)	
E2 (ε2/ε2, ε2/ε3)	16.7
E3 (ε3/ε3)	66.2
E4 (ε2/ε4, ε3/ε4, ε4/ε4)	17.1
Age (years, median [IQR])	83 [5]
MMSE score (median [IQR])	29 [1]
Clinical diagnosis (%)	
CU	89.5
MCI	10.5
Education (CASMIN classification, %)	
Elementary	58.1
Intermediate	29.4
High	12.5
BMI (median [IQR])	25.6 [4.7]
Hypercholesterolemia (%)	61.2
Diabetes (%)	23.9
Heart insufficiency (%)	25.0
eGFR (mL/min/BSA)	58 [22]
Plasma biomarkers	
Aβ42/Aβ40 ratio (median [IQR])	0.1 [0.02]
P-tau181 (pg/mL, median [IQR])	2.8 [1.7]
NfL (pg/mL, median [IQR])	31.9 [18.1]
GFAP (pg/mL, median [IQR])	120 [75.5]

IQR, interquartile range; CU, cognitively unimpaired; MCI, mild cognitive impairment; BMI, body mass index; eGFR, estimated glomerular filtration rate; BSA, body surface area.

Throughout the observational period of approximately 8 years, 17.7% of the participants developed clinically-diagnosed DAT, while 4.6% experienced other forms of dementia. A 26.1% died without developing clinically-diagnosed DAT during this period. The median FU time for participants who did not progress to clinically-diagnosed DAT was 7.2 years, with the median duration until progression to clinically-diagnosed DAT being 4.5 years. Plasma levels of P-tau181, NfL, and GFAP were significantly correlated with age, but the correlation with P-tau181 was very weak (P-tau181: r = 0.07, *p* = 0.02; NfL: r = 0.15, *p* = 1.37 × 10^−6^; GFAP: r = 0.21, *p* = 2.62 × 10^−11^). Aβ42/Aβ40 ratio was not correlated (r = 0.04, *p* = 0.26) (Supplementary Figure S1).

### Plasma biomarkers are associated with cognitive decline and progression to clinically-diagnosed dementia of the Alzheimer's type

**Fig. 2:**
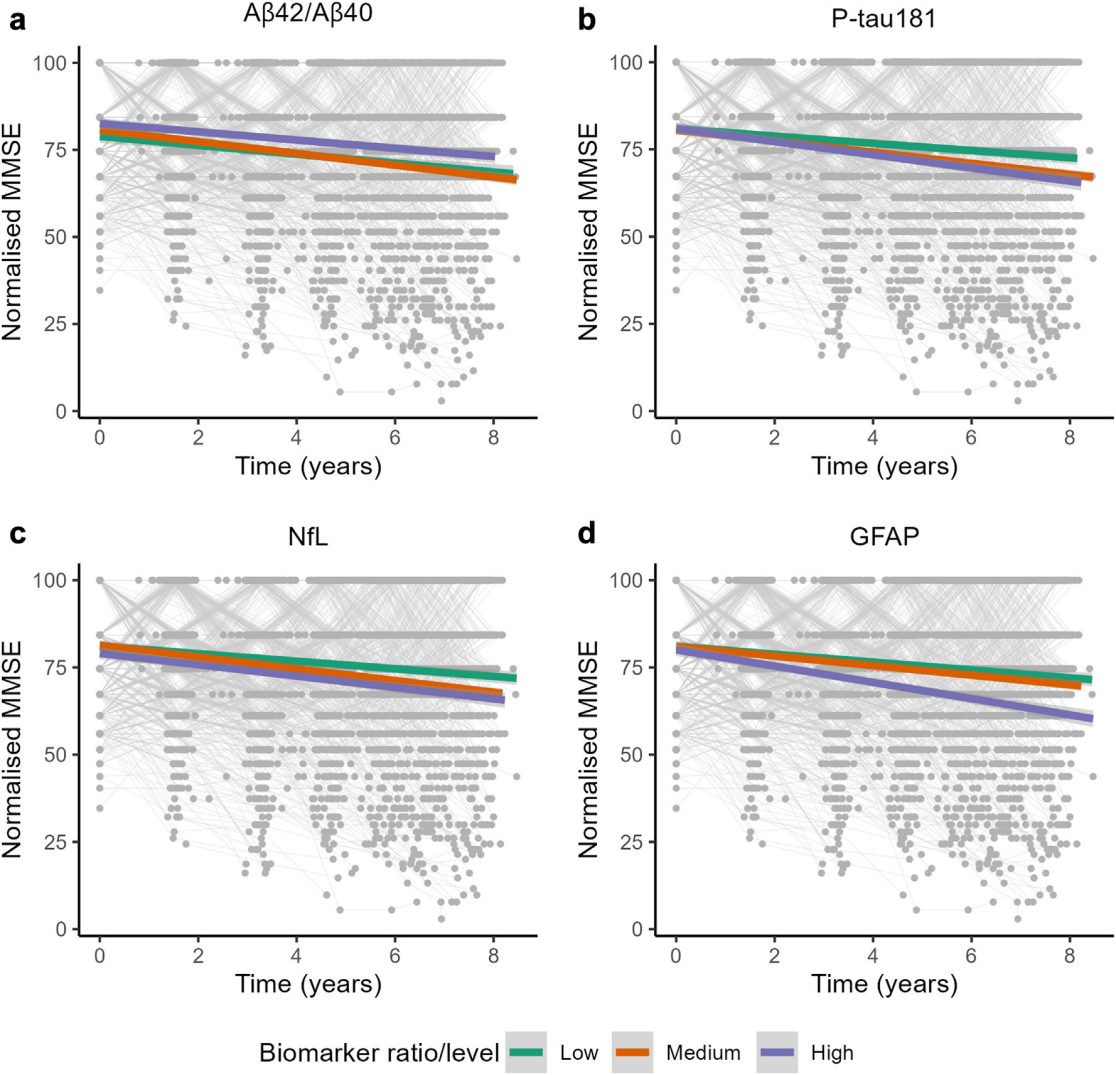
**Association between baseline plasma biomarker ratio or levels and cognitive decline in participants without dementia.** Spaghetti plots show the association between plasma biomarker ratio/levels and cognitive decline for each participant without dementia. Normalised MMSE score was used as a proxy of cognitive performance. Biomarkers were split into quantiles with low (green, below 25%), medium (orange, 25–75%), and high (purple, over 75%) ratio/levels and regression lines were fitted for each group only for visualisation purposes. Each circle represents a MMSE score measurement. (a) Aβ42/Aβ40, N = 827; (b) P-tau181, N = 915; (c) NfL, N = 924; (d) GFAP, N = 925.

We first explored whether baseline plasma biomarkers were associated with cognitive decline across all participants. The linearity of the normalised MMSE trajectories, a measure of cognitive function, was evaluated by comparing two models: a) a linear mixed-effects model comprising solely the fixed effect of time (longitudinal MMSE ∼ time), a random intercept, and a random slope of time, and b) a second model incorporating both the fixed effects of time and (time)^2^ (longitudinal MMSE ∼ time + (time)^2^), a random intercept, a random slope of time, and a random slope of (time)^2^. Despite both models demonstrating very similar Bayesian Information Criterion (BIC) values (model a: BIC = 44,002; model b: BIC = 43,987), we opted for the more parsimonious model a), which included only the fixed effect of time. Then, we performed linear mixed-effect models for each biomarker using the interaction between time elapsed and biomarker ratio/level, age, sex, *APOE* strata, and education levels as predictors, and normalised MMSE score as the outcome cognitive measure (longitudinal MMSE ∼ time + biomarker + age + sex + *APOE* + education + time x biomarker + time x age + time x sex + time x APOE + time x education). We found that higher baseline plasma levels of P-tau181 (β = −0.49, *p* = 2.53 × 10^−5^), NfL (β = −0.29, *p* = 0.01), and GFAP (β = −0.60, *p* = 1.99 × 10^−7^) were associated with faster cognitive decline, but no association was found with baseline plasma Aβ42/Aβ40 ratio (β = 0.10, *p* = 0.40) (Fig. 2, Supplementary Table S1). To assess whether these findings might be influenced by individuals in the prodromal stage, we excluded participants with MCI, thereby examining this association specifically in individuals without cognitive impairment. Similarly, we observed that higher baseline plasma levels of P-tau181 (β = −0.42, *p* = 5.45 × 10^−4^), NfL (β = −0.26, *p* = 0.03), and GFAP (β = −0.52, *p* = 1.38 × 10^−5^) were associated with faster cognitive decline, but not baseline Aβ42/Aβ40 ratio (β = 0.11, *p* = 0.35) (Supplementary Table S2, Supplementary Figure S2).

Next, to evaluate the association between plasma biomarkers and risk of progressing to clinically-diagnosed DAT in participants without dementia, we performed Cox regressions adjusted for age, sex, and eGFR. We observed that higher levels of P-tau181 (HR = 1.32, *p* = 1.73 × 10^−5^), NfL (HR = 1.19, *p* = 0.01), and GFAP (HR = 1.36, *p* = 9.83 × 10^−8^), and a lower Aβ42/Aβ40 ratio (HR = 0.80, *p* = 8.91 × 10^−3^) were associated with a higher risk (Table 2). Then, we repeated the same analysis excluding participants with MCI. In line with the results from participants without dementia, we found that higher levels of P-tau181 (HR = 1.34, *p* = 3.21 × 10^−5^) and GFAP (HR = 1.32, *p* = 2.09 × 10^−5^), and a lower Aβ42/Aβ40 ratio (HR = 0.78, *p* = 0.01) were associated with a higher risk. However, NfL was not significantly associated (HR = 1.14, *p* = 0.09) (Supplementary Table S3). Finally, we investigated the association between plasma biomarkers and risk of progressing to MCI. Interestingly, we observed that higher levels of P-tau181 and GFAP were associated with an increased risk (P-tau181: HR = 1.16, *p* = 8.03 × 10^−3^; GFAP: HR = 1.20, *p* = 4.43 × 10^−4^), while Aβ42/Aβ40 ratio and NfL were not (Aβ42/Aβ40: HR = 0.90, *p* = 0.11; NfL: HR = 1.04, *p* = 0.54) (Supplementary Table S4).

**Table 2:** Association between baseline plasma biomarker ratio or levels and risk of progressing to dementia of the Alzheimer's type in participants without dementia.

Variables	Aβ42/Aβ40 N = 861			P-tau181 N = 950			NfL N = 960			GFAP N = 961		
	HR	95% CI	*p*-value	HR	95% CI	*p*-value	HR	95% CI	*p*-value	HR	95% CI	*p*-value
Biomarker	0.80	0.68–0.95	8.91 × 10^−3^	1.32	1.17–1.51	1.73 × 10^−5^	1.19	1.03–1.36	0.01	1.36	1.22–1.52	9.83 × 10^−8^
Age	1.53	1.32–1.76	8.76 × 10^−9^	1.50	1.31–1.71	2 × 10^−9^	1.48	1.29–1.69	9.31 × 10^−9^	1.41	1.23–1.61	6.33 × 10^−7^
Sex (male)	0.59	0.40–0.86	6.94 × 10^−3^	0.48	0.33–0.70	1.36 × 10^−4^	0.57	0.40–0.82	2.38 × 10^−3^	0.63	0.43–0.90	0.01
eGFR	0.97	0.83–1.15	0.75	1.11	0.95–1.30	0.18	1.08	0.92–1.27	0.35	1.07	0.92–1.24	0.40

eGFR, estimated glomerular filtration rate; HR, hazard ratio; CI, confidence interval. All continuous variables were *z*-transformed to allow comparison.

### Plasma biomarkers improve prediction of future clinically-diagnosed dementia of the Alzheimer's type

Given that plasma biomarkers were associated with cognitive decline and disease progression in participants without dementia, we decided to assess whether they could help improve the DAT diagnosis that could be achieved at the primary care level using only age, sex, and *APOE* genotype. To this end, we followed the recommendations of the EU/US CTAD Task Force and Global CEO Initiative on Alzheimer's regarding the use of plasma biomarkers by primary care providers for triaging and identifying patients who would benefit most from therapy.^19^^,^^20^ Therefore, we evaluated the positive (PPV), negative predictive values (NPV), sensitivity, and specificity in three distinct scenarios over a four-year period: a) solely incorporating a brief cognitive assessment (MMSE score), b) exclusively introducing plasma biomarkers, and c) including both MMSE score and plasma biomarkers. The use of PPV and specificity as metrics holds particular significance in this context, given the elevated cost associated with false positives. In the first scenario, MMSE displayed a significant association with clinically-diagnosed DAT progression status conditional on age, sex, and *APOE* genotype (*p* = 2.79 × 10^−8^), yielding a model PPV of 0.20 and a specificity of 0.60. In the second scenario, substituting MMSE score with plasma biomarkers showed similar metrics (PPV = 0.21, specificity = 0.66), with only plasma Aβ42/Aβ40 ratio (*p* = 0.03) and GFAP level (*p* = 0.01) significantly associated with clinically-diagnosed DAT progression status. In the third scenario, both MMSE and GFAP level exhibited a significant association with clinically-diagnosed DAT progression status (MMSE, *p* = 1.13 × 10^−7^; GFAP, *p* = 0.02), with a PPV of 0.21 and a specificity of 0.62. All models displayed an NPV equal or higher than 0.95 and sensitivities over 0.70 (Table 3, Supplementary Table S5).

**Table 3:** Performance of different strategies to predict future dementia of Alzheimer's type.

Strategy	Model parameters								
	TP	FP	TN	FN	PPV	NPV	Sens	Spec	AUC
All participants (N = 809; DAT progression, N = 89)									
Age + sex + *APOE* strata	70	385	335	19	0.15	0.95	0.79	0.47	0.67
MMSE + age + sex + *APOE* strata	73	289	431	16	0.20	0.96	0.82	0.60	0.75
Biomarkers + age + sex + *APOE* strata	66	248	472	23	0.21	0.95	0.74	0.66	0.72
MMSE + biomarkers + age + sex + *APOE* strata	71	272	448	18	0.21	0.96	0.80	0.62	0.77
Participants with MMSE≤27 (N = 192; DAT progression, N = 45)									
Age + sex + *APOE* strata	36	72	75	9	0.33	0.89	0.80	0.51	0.65
MMSE + age + sex + *APOE* strata	27	41	106	18	0.40	0.85	0.60	0.72	0.71
Biomarkers + age + sex + *APOE* strata	35	49	98	10	0.42	0.91	0.78	0.67	0.74
MMSE + biomarkers + age + sex + *APOE* strata	27	26	121	18	0.51	0.87	0.60	0.82	0.75

DAT, clinically-diagnosed dementia of the Alzheimer's type; TP, true positive cases; FP, false positive cases; TN, true negative cases; FN, false negative cases; PPV, positive predictive value; NPV, negative predictive value; Sens, sensitivity; Spec, specificity; AUC, area under the ROC curve.

Since all scenarios displayed low PPV and specificity, and thus a large number of false positives, we decided to test the same models but in a subset of patients with suspected MCI based only on a brief cognitive assessment, adopting a two-step strategy. Therefore, we selected all participants with an MMSE score ≤27,^21^ and evaluated the PPV and specificity again a) solely incorporating MMSE score, b) exclusively introducing plasma biomarkers, and c) including both MMSE score and plasma biomarkers. By enriching the sample in participants with suspected MCI, we observed an improvement in PVV and specificity in all models. The first one exhibited a PPV of 0.40 and a specificity of 0.72, and MMSE score was significantly associated with DAT progression status (*p* = 0.01). Exclusively introducing biomarkers yielded a PPV of 0.42 and a specificity of 0.67, with only plasma Aβ42/Aβ40 ratio significantly associated with clinically-diagnosed DAT progression status (*p* = 0.02). Notably, incorporating both MMSE score and plasma biomarkers exhibited the highest PPV (0.51) and specificity (0.82), with only MMSE score (*p* = 0.01) and plasma Aβ42/Aβ40 (*p* = 0.02) ratio significantly associated with clinically-diagnosed DAT progression status. All models displayed an NPV equal or higher than 0.85 and lower sensitivities when compared to the model performance that included all individuals (Table 3, Supplementary Table S5).

Next, we sought to determine whether measuring all plasma biomarkers or only a subset was necessary for optimal predictive performance. To this end, we evaluated models b) and c) using only plasma Aβ42/Aβ40 ratio and GFAP level in all participants or in those with an MMSE score ≤27. We included only plasma Aβ42/Aβ40 ratio and GFAP level since they were the only ones significantly associated with clinically-diagnosed DAT progression status in the models including all biomarkers. Interestingly, the models excluding plasma levels of P-tau181 and NfL performed similarly to those including all biomarkers (Supplementary Tables S6 and S7). To further simplify the models, we also tested a model with only plasma Aβ42/Aβ40 ratio and GFAP level, and another one with MMSE plus plasma Aβ42/Aβ40 ratio and GFAP level. Both models excluded basic demographics and *APOE* stratum to explore the performance of biomarkers or biomarkers plus MMSE alone. However, we found that both PPV and specificity worsened in these simplified models (Supplementary Tables S6 and S7). These results suggest that a combination of brief cognitive testing, plasma Aβ42/Aβ40 ratio and GFAP level, along with age, sex, and *APOE* stratum, could help primary care providers better identify patients eligible for treatment among those with evidence of cognitive impairment.

### Plasma biomarker levels vary over time

A longitudinal strategy, which includes repeated biomarker measurements in patients without dementia, could prove more beneficial for monitoring and prognosis than depending on a value obtained in a single time-point measurement. Consequently, we decided to estimate the trajectories of plasma biomarkers from the participants who also had an FU sample (N = 305, ∼8 years later). From this subset of participants, 64.3% were female and 15.6% had at least a copy of an *APOE*-ε4 allele. The median age at baseline was 82 with a median MMSE score of 29. The majority of the participants did not develop dementia (without cognitive impairment, 69.2%; MCI, 14.8%), and 14.8% progressed to clinically-diagnosed DAT. Most of the participants had elementary education (55.1%), 26.9% intermediate level, and 18% a high level. At baseline, the median BMI was 25.6 and the median eGFR 59, and 61.3% of the participants were diagnosed with hypercholesterolemia, 20.2% with diabetes, and 15.6% with heart insufficiency (Table 4). Plasma levels of NfL and GFAP were significantly correlated with age both at baseline and FU (baseline, NfL: r = 0.21, *p* = 3.16 × 10^−4^; GFAP: r = 0.22, *p* = 1.03 × 10^−4^; FU, NfL: r = 0.33, *p* = 3.54 × 10^−9^; GFAP: r = 0.21, *p* = 2.18 × 10^−4^) (Supplementary Figures S3 and S4).

**Table 4:** Clinical and demographic characteristics of participants without dementia at baseline with a follow-up sample.

Characteristics	Baseline (N = 305)	FU (N = 305)
Sex (Female, %)	64.3	64.3
*APOE* strata (%)		
E2 (ε2/ε2, ε2/ε3)	16.7	16.7
E3 (ε3/ε3)	67.7	67.7
E4 (ε2/ε4, ε3/ε4, ε4/ε4)	15.6	15.6
Age (years, median [IQR])	82 [4]	90 [4]
MMSE score (median [IQR])	29 [2]	28 [3]
Clinical diagnosis (%)		
CU	93.1	69.2
MCI	6.9	14.8
DAT	–	14.8
Vascular dementia	–	1.3
Education (CASMIN classification, %)		
Elementary	55.1	55.1
Intermediate	26.9	26.9
High	18	18
BMI (median [IQR])	25.6 [4.6]	25.1 [5]
Hypercholesterolemia (%)	61.3	51.7
Diabetes (%)	20.2	28.2
Heart failure (%)	15.6	36.9
eGFR (mL/min/BSA)	59 [21]	–
Plasma biomarkers		
Aβ42/Aβ40 ratio (median [IQR])	0.1 [0.02]	0.1 [0.02]
P-tau181 (pg/mL, median [IQR])	2.7 [1.6]	3.9 [2.4]
NfL (pg/mL, median [IQR])	29.9 [17.9]	47.9 [35.7]
GFAP (pg/mL, median [IQR])	114 [68]	176.5 [118]

FU, follow-up; IQR, interquartile range; CU, cognitively unimpaired; MCI, mild cognitive impairment; DAT, clinically-diagnosed dementia of the Alzheimer's type; BMI, body mass index; eGFR, estimated glomerular filtration rate; BSA, body surface area.

**Fig. 3:**
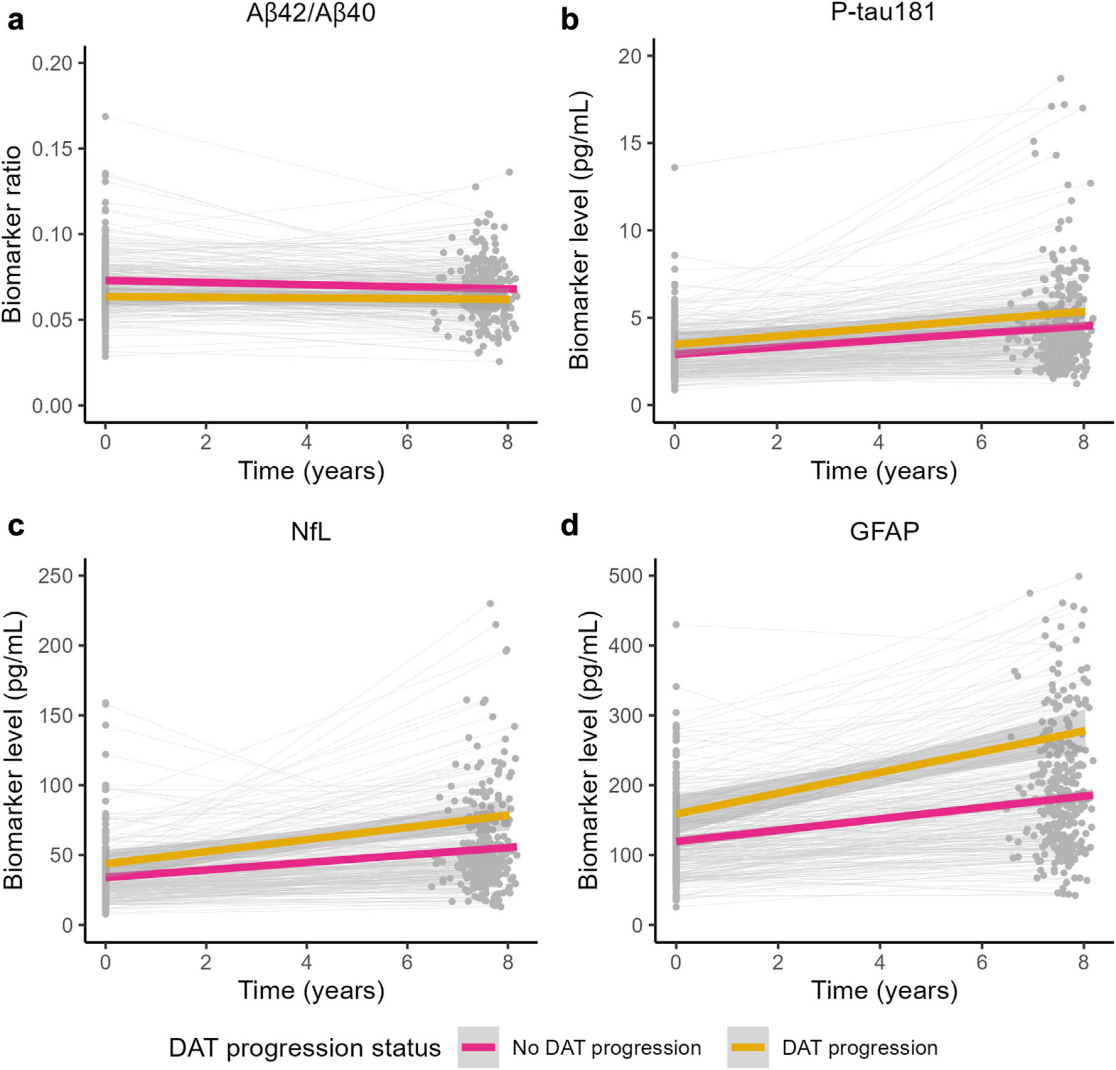
**Dynamics of plasma biomarkers in relation to clinical diagnosis.** Spaghetti plots show the change in biomarker ratio/level from baseline until follow-up in participants that progressed to clinically-diagnosed dementia of the Alzheimer's type (yellow, DAT progression) or remained without dementia or progressed to other types of dementia (pink, no DAT progression). Each circle represents a biomarker measurement. Regression lines were fitted for each group only for visualisation purposes. (a) Aβ42/Aβ40, N = 283; (b) P-tau181, N = 284; (c) NfL, N = 287; (d) GFAP, N = 287. DAT, clinically-diagnosed dementia of the Alzheimer's type.

To evaluate the dynamics of each biomarker, we performed linear mixed-effect models for repeated measures using the interaction term between time elapsed and age, sex, *APOE* strata, eGFR, and clinically-diagnosed DAT progression status as predictors, and plasma biomarker ratio/level as the outcome measure. We observed changes in all biomarkers over time, with an elevation in P-tau181 (β = 0.19, *p* = 1.83 × 10^−11^), NfL (β = 2.88, *p* = 2.44 × 10^−14^), and GFAP (β = 8.23, *p* = 1.60 × 10^−22^) levels, and a reduction in Aβ42/Aβ40 ratio (β = −6.89 × 10^−4^, *p* = 1.54 × 10^−3^), even though the small effect size for the latter was negligible. NfL was the sole biomarker that demonstrated an age-associated increase in levels (β = 0.96, *p* = 1.61 × 10^−4^). Interestingly, among those participants who progressed to clinically-diagnosed DAT during the FU period, NfL (β = 2.47, *p* = 7.38 × 10^−4^) and GFAP (β = 4.45, *p* = 4.59 × 10^−3^) levels exhibited a more rapid increase, while Aβ42/Aβ40 ratio and P-tau181 variation remained independent of the clinically-diagnosed DAT progression status (Aβ42/Aβ40: β = 6.18 × 10^−4^, *p* = 0.18; P-tau181: β = 9.74 × 10^−3^, *p* = 0.86) (Fig. 3, Supplementary Table S8).

## Discussion

AD begins 20 or more years before symptoms arise, giving a substantial time window for intervention. The general belief is that treating AD at an early stage is likely to result in more clinically meaningful effects.^22^, ^23^, ^24^ Primary care appears as the natural setting for early detection because the majority of the population at risk visits the GPs at some point. In this large prospective primary care-based study, we evaluated the prognostic performance of plasma Aβ42/Aβ40 ratio and plasma levels of P-tau181, NfL, and GFAP, and confirmed that these biomarkers are associated with an increased risk of progressing to clinically-diagnosed DAT during ∼8 years of follow-up in individuals without dementia, aged 79–94 years, and regularly visiting the primary care system. In addition, while higher plasma levels of P-tau181, NfL, and GFAP were associated with faster cognitive decline, a lower plasma Aβ42/Aβ40 ratio was not. We also observed that plasma Aβ42/Aβ40 ratio and GFAP level, together with MMSE score, improved the diagnosis precision of future clinically-diagnosed DAT in participants with a lower MMSE score (≤27). Finally, in a subset of patients with a matched sample after ∼8 years of follow-up, all plasma biomarkers showed time-dependent changes that should be considered when assessing the risk of progressing to clinically-diagnosed DAT.

Although the association between plasma biomarkers and cognitive decline, as well as the risk of progressing to DAT, has been extensively examined in clinically-based cohorts,^8^^,^^25^^,^^26^ to our knowledge no studies had yet extensively studied Aβ42/Aβ40 ratio, P-tau181, NfL, and GFAP in the oldest old individuals without dementia in primary care. Our findings indicate that plasma levels of P-tau181, NfL, and GFAP significantly contribute to predicting cognitive decline and the risk of progressing to clinically-diagnosed DAT, while Aβ42/Aβ40 ratio appears to be more useful in predicting the risk of progression to clinically-diagnosed DAT. This observation suggests that Aβ42/Aβ40 ratio is probably an early marker of AD pathology that reaches a plateau already at the asymptomatic stage of AD, before changes leading to cognitive symptoms and decline become evident.^27^ Interestingly, the association between plasma NfL and risk of progressing to clinically-diagnosed DAT became non-significant when the analyses were run including only individuals without cognitive impairment, suggesting that plasma NfL, unlike Aβ42/Aβ40 ratio, becomes altered when cognitive decline is noticeable. Consistent with these findings, a prior population-based study revealed a decline in the association between NfL and DAT with increasing FU duration.^28^ Additionally, a recent memory clinic study indicated that a higher baseline plasma NfL level was not linked to a more pronounced rate of cognitive decline.^8^

The Alzheimer's Association identified the number of available appointments to see dementia specialists in the US as a primary bottleneck, contributing to an estimated average wait time of 19 months between seeking diagnosis and initiating a disease-modifying Alzheimer's treatment therapy (DMT).^29^ Therefore, improving triage at the primary care level seems a logical approach to reduce the expected wait time. It has been proposed that conducting a blood-based biomarker test in primary care patients with suspected MCI, based on a brief cognitive assessment, would dramatically improve the efficiency of the initial evaluation process.^19^^,^^30^ In our study, we observed that in participants with an MMSE score ≤27, combining plasma biomarkers, MMSE score, age, sex, and *APOE* stratum into a model to predict progression to clinically-diagnosed DAT resulted in a PPV of 0.51 and a specificity of 0.82. This specificity is close to the ideal ≥0.85 recommended by the Global CEO Initiative on Alzheimer's Disease for a triaging test in primary care to identify individuals with an increased likelihood of having amyloid pathology, who would then require confirmatory testing with amyloid PET or CSF.^20^ Hence, employing a two-step approach could help minimise costs and wait times and enhance the identification of true-positive cases, since specialists in memory disorders would be relieved from the task of assessing numerous false-positive referrals. Nevertheless, this strategy comes at the expense of lower sensitivity (0.60), increasing the number of false-negative results, which could lead to delays in care or misdiagnosis. This may be due to patients having a borderline level of amyloid pathology, with values near the cut-off. This drawback could be mitigated by using a two-cut-off strategy, as recently recommended,^20^ which defines three categories of results: positive, intermediate, and negative, thereby increasing the overall accuracy. Patients with a value near the cut-off would fall into the intermediate category, and the most appropriate approach would depend on the patient and settings. If the patient is a potential candidate for DMT, a confirmatory amyloid PET or CSF test would be required. If not, scheduling follow-up appointments with the GP, where the plasma biomarkers test could be repeated, might be reasonable. Notably, out of all plasma biomarkers, only Aβ42/Aβ40 ratio and GFAP level exhibited a noteworthy association with clinically-diagnosed DAT progression status, even when accounting for MMSE score and demographic factors. This suggests that while plasma biomarkers offer overlapping information, Aβ42/Aβ40 ratio and GFAP stand out as particularly relevant. Consistent with these findings, a prior investigation demonstrated that when combining plasma Aβ42/Aβ40 ratio with plasma levels of P-tau217 and NfL in a predictive model for DAT progression among individuals without cognitive impairment, only Aβ42/Aβ40 ratio and P-tau217 contributed independent predictive information.^31^

Evaluating the dynamic changes of plasma biomarkers in the oldest-old population, as opposed to depending on a single measurement at a specific time-point, may offer greater utility for following-up patients in the preclinical stage and evaluating prognosis in patients in the prodromal stage. Our results of biomarker trajectories show that after eight years, plasma levels of P-tau181, NfL, and GFAP exhibited a time-related elevation. Moreover, individuals who developed clinically-diagnosed DAT during the observational period demonstrated a more rapid increase in plasma levels of NfL and GFAP. Notably, the change in plasma P-tau181 level between conversion status groups revealed no significant differences, as previously reported.^28^^,^^32^ Interestingly, age had a significant effect on NfL concentration over time, confirming previous results^33^ and suggesting that age should be taken into account when defining cut-offs for this biomarker. These findings underscore the potential of NfL and GFAP in serving as valuable tools for monitoring disease severity and as robust outcome measures in clinical trials.

Our study also has limitations. First, DAT diagnosis was clinically-based. We were unable to confirm AD pathological diagnosis because neither CSF samples nor PET scans were available in the primary care-based AgeCoDe study. Second, the implementation of plasma biomarkers in primary care will require additional education and training for GPs on how to assess people with cognitive impairment and how to interpret plasma biomarkers results. Untrained GPs could interpret that an individual with a negative or borderline plasma biomarker result does not need further assessment, or disregard other co-existing pathologies in case of a positive biomarker result. Third, MMSE exhibits some challenges as a screening tool, such as difficulty in identifying mild cognitive impairments and interpreting the results, as age, education, and cultural background affect scores. Fourth, we cannot rule out variations in biomarker concentration due to suboptimal pre-analytics or storage time, even though our results are in line with previous population and memory clinic-based studies. Fifth, our analyses included only Aβ42/Aβ40 ratio, P-tau181, NfL, and GFAP, as P-tau217, a biomarker that can accurately identify most amyloid-positive individuals,^34^ was not available in our dataset. Finally, we were able to evaluate predictive performance only within our primary care sample. Replication of our models in independent, longitudinal cohorts is required to confirm our findings.

In conclusion, our results revealed a significant association between plasma Aβ42/Aβ40 ratio, P-tau181, NfL, and GFAP levels and the risk of progressing to DAT in patients of advanced age visiting primary care. Particularly, Aβ42/Aβ40 ratio exhibited an early indicative role, while NfL could emerge as a later-stage marker. Including Aβ42/Aβ40 ratio and GFAP level improved the specificity for identifying individuals with evidence of cognitive impairment who progressed to clinically-diagnosed DAT, compared to using only MMSE score and basic demographics. Repeated measurements suggested that plasma NfL and GFAP, while lacking AD specificity, hold potential for effectively monitoring progression to DAT. Moving forward, it will be critical to demonstrate the effectiveness of plasma biomarkers when used prospectively and longitudinally with predefined cut-offs in real-world populations with ethnocultural diversity. Additionally, there is a crucial need to control pre-analytical, analytical, and post-analytical sources of variability. Taken together, these results demonstrate that, if implemented effectively and efficiently, the evaluation of multiple plasma biomarkers, coupled with brief cognitive assessments, promises a substantial enhancement in the precision of risk assessment for DAT progression within primary care settings, thus reducing the current bottleneck for secondary care assessment.

## Contributors

PVMA designed and conceptualised the study, analysed and interpreted the data, contributed to data acquisition, and wrote the manuscript. MC, KB, HZ, MP, SW, HB, BW, SGRH, and MS contributed to data acquisition. LK and MW contributed to data interpretation. AS contributed to data acquisition and interpretation. AR designed and conceptualised the study, interpreted the data, contributed to data acquisition, and wrote the manuscript.

## Data sharing statement

Anonymised clinical data and samples from AgeCoDe study will be shared upon request in the form of research collaborations, as long as it complies with GDPR legislation and it is approved by AgeCoDe steering committee. The code utilised for statistical analysis can be provided by the corresponding author upon inquiry.

## Declaration of interests

HZ has served at scientific advisory boards and/or as a consultant for Abbvie, Acumen, Alector, Alzinova, ALZPath, Amylyx, Annexon, Apellis, Artery Therapeutics, AZTherapies, Cognito Therapeutics, CogRx, Denali, Eisai, Merry Life, Nervgen, Novo Nordisk, Optoceutics, Passage Bio, Pinteon Therapeutics, Prothena, Red Abbey Labs, reMYND, Roche, Samumed, Siemens Healthineers, Triplet Therapeutics, and Wave, has given lectures in symposia sponsored by Alzecure, Biogen, Cellectricon, Fujirebio, Lilly, Novo Nordisk, and Roche, and is a co-founder of Brain Biomarker Solutions in Gothenburg AB (BBS), which is a part of the GU Ventures Incubator Program (outside submitted work). KB has served as a consultant and at advisory boards for AC Immune, Acumen, ALZPath, AriBio, BioArctic, Biogen, Eisai, Lilly, Moleac Pte. Ltd, Novartis, Ono Pharma, Prothena, Roche Diagnostics, and Siemens Healthineers; has served at data monitoring committees for Julius Clinical and Novartis; has given lectures, produced educational materials and participated in educational programs for AC Immune, Biogen, Celdara Medical, Eisai and Roche Diagnostics; and is a co-founder of Brain Biomarker Solutions in Gothenburg AB (BBS), which is a part of the GU Ventures Incubator Program, outside the work presented in this paper. AS received honorarium for a lecture by Eisai and participates in the scientific advisory board of the Biogen CELIA study. MS is the current president of the German Society of General Practice and Family Medicine (DEGAM). The other authors report no conflict of interests.

## Appendix A Supplementary data

Supplementary Lancet Reg Health
