# Supplementary material for: Prognostic value of Alzheimer's disease plasma biomarkers in the oldest-old: a prospective primary care-based study
Source: Lancet Reg Health Eur. 2024 Aug 16;45:101030. doi: 10.1016/j.lanepe.2024.101030 (PMC11381503; doi:10.1016/j.lanepe.2024.101030)
Supplement: Supplementary Lancet Reg Health [file mmc1.docx]

**Supplementary material**

**Study participants.** The German Study on Ageing, Cognition, and Dementia (AgeCoDe) is a multicentre prospective cohort study of German patients in primary care. Participants were recruited from 2003 to 2004 in six German cities (Bonn, Düsseldorf, Hamburg, Leipzig, Mannheim, and Munich) by 19–29 general practitioners (GPs) per site (138 in total), representing an urban area of cities with a total population ranging between about 300000 (Mannheim) and almost 1.8 million (Hamburg).^1,2^ Inclusion criteria for potentially eligible patients were at least 75 years of age, absence of dementia according to the judgement of the GP, and at least one contact with the GP within the past 12 months. Exclusion criteria were consultations only by home visits, severe illness, insufficient knowledge of German language, deafness or blindness, and lack of capability for informed consent. Since a medical record registry approach was used rather than a waiting room recruitment strategy, the participants in this study are unselected and can be considered representative of community-dwelling older adults. Of the 10850 eligible patients, 6619 randomly selected patients were contacted by mail, and 3327 gave informed consent to the GP for participation and received follow-up assessments every 18 months (at baseline and in nine follow-up assessments). All assessments were performed by trained physicians, psychologists, and gerontologists at the patient’s home environment using standardised questionnaires. Apart from these patient interviews, GPs provided information regarding their participating patients and thus the entire cohort was continuously monitored for morbidity and mortality. Information on the cognitive status of those who had died in the interim was collected from family members, caregivers, or GPs. The present study was approved by the respective ethics committees, and written informed consent was obtained from all participants before inclusion. All study procedures complied with national legislation and the Code of Ethical Principles for Medical Research Involving Human Subjects of the World Medical Association.

**Neuropsychological assessments.** Neuropsychological and clinical assessments were based on the Structured Interview for Diagnosis of Dementia of Alzheimer type, Multi-infarct dementia and dementia of other aetiology according to DSM-IV and ICD-10 (SIDAM),^3^ and the semantic verbal fluency test and the verbal memory test (10-item word list) of the neuropsychological battery of the Consortium to Establish a Registry for Alzheimer's Disease (CERAD).^4^ Dementia status in patients without personal interview at follow-up (for example, because of refusal or death) was based on additional informant interviews with relatives, professional carers, and GPs, using the Global Deterioration Scale (GDS)^5^ and Blessed Dementia Scale.^6^ All diagnoses were discussed in consensus conferences with the interviewers and experienced geriatric psychiatrists or geriatricians. Dementia diagnoses could be verified by follow-up results. MCI was defined according to one standard deviation deficit in any cognitive domain in the cognitive test battery implemented in the SIDAM.

**Education.** The German education system is highly diverse, resulting in a lack of linear correlation between the duration of schooling and the level of education attained. For this reason, the level of education received by the patients was measured by the highest completed level and categorised as low (primary school level/elementary education), intermediate (intermediate vocational/general qualification), or high (undergraduate or postgraduate studies/tertiary education) using the Comparative Analysis of Social Mobility in Industrial Nations (CASMIN) international educational classification.^7^ The CASMIN classification distinguishes between hierarchically structured educational qualifications and provides international comparability.

**
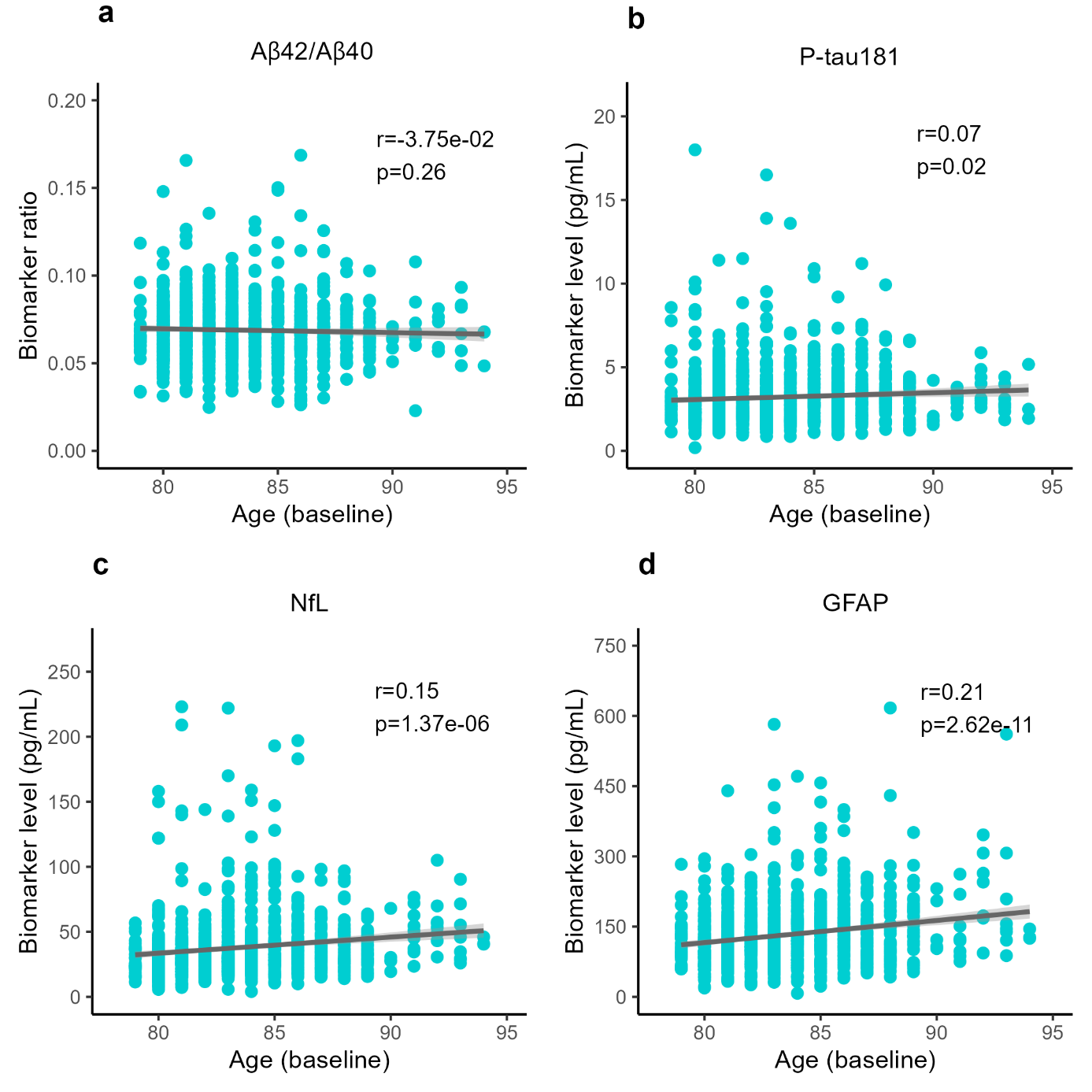
**

**Supplementary Figure 1. Correlation of baseline plasma biomarkers with age in participants without dementia.** Scatter plots show the association between age at baseline and plasma Aβ42/Aβ40 ratio (a), or plasma levels of P-tau181 (b), NfL (c), and GFAP (d). r, Pearson correlation coefficient.


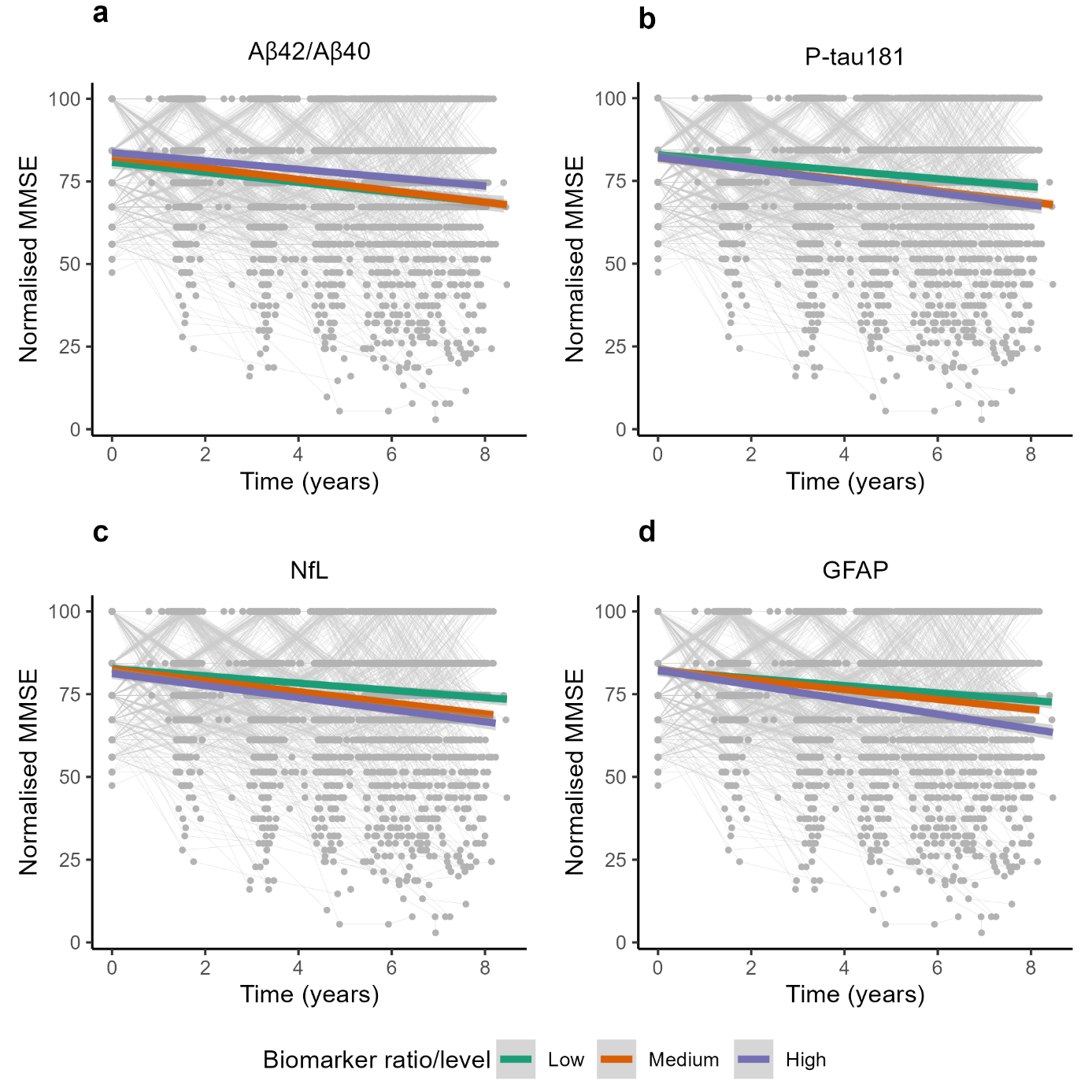


**Supplementary Figure 2. Association between baseline plasma biomarker levels and cognitive decline in participants without cognitive impairment.** Spaghetti plots show the association between plasma biomarker levels and cognitive decline for each participant without cognitive impairment. Normalised MMSE score was used as a proxy of cognitive performance. Biomarkers were split into quantiles with low (below 25%), medium (25-75%), and high (over 75%) ratio/levels and trend lines were fitted for each group only for visualisation purposes. Aβ42/Aβ40, N=743; P-tau181, N=823; NfL, N=834; GFAP, N=836.


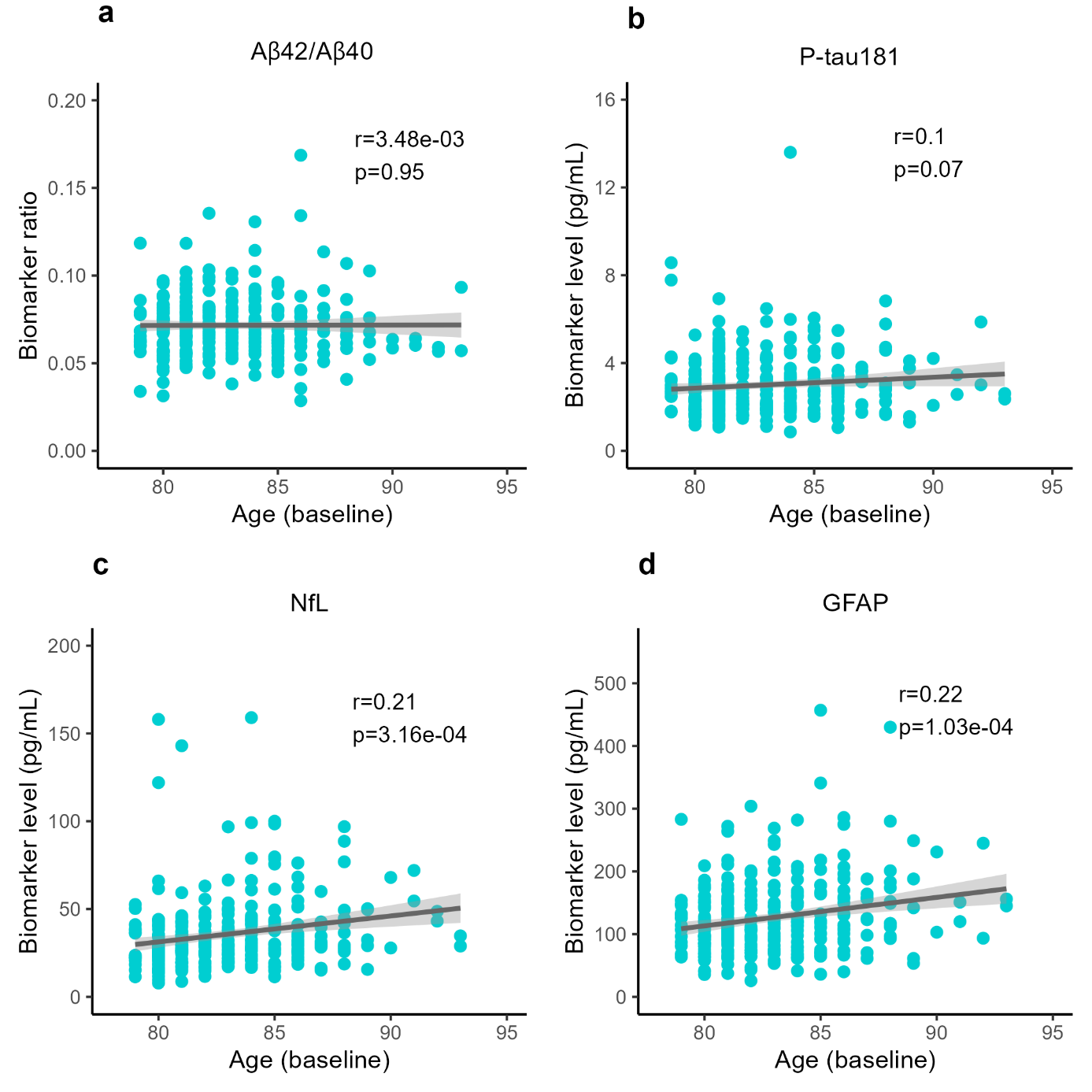


**Supplementary Figure 3. Correlation of baseline plasma biomarkers with age in the subset of participants without dementia with a follow-up sample.** Scatter plots show the association between age at baseline and plasma Aβ42/Aβ40 ratio (a), or plasma levels of P-tau181 (b), NfL (c), and GFAP (d). r, Pearson correlation coefficient.

**
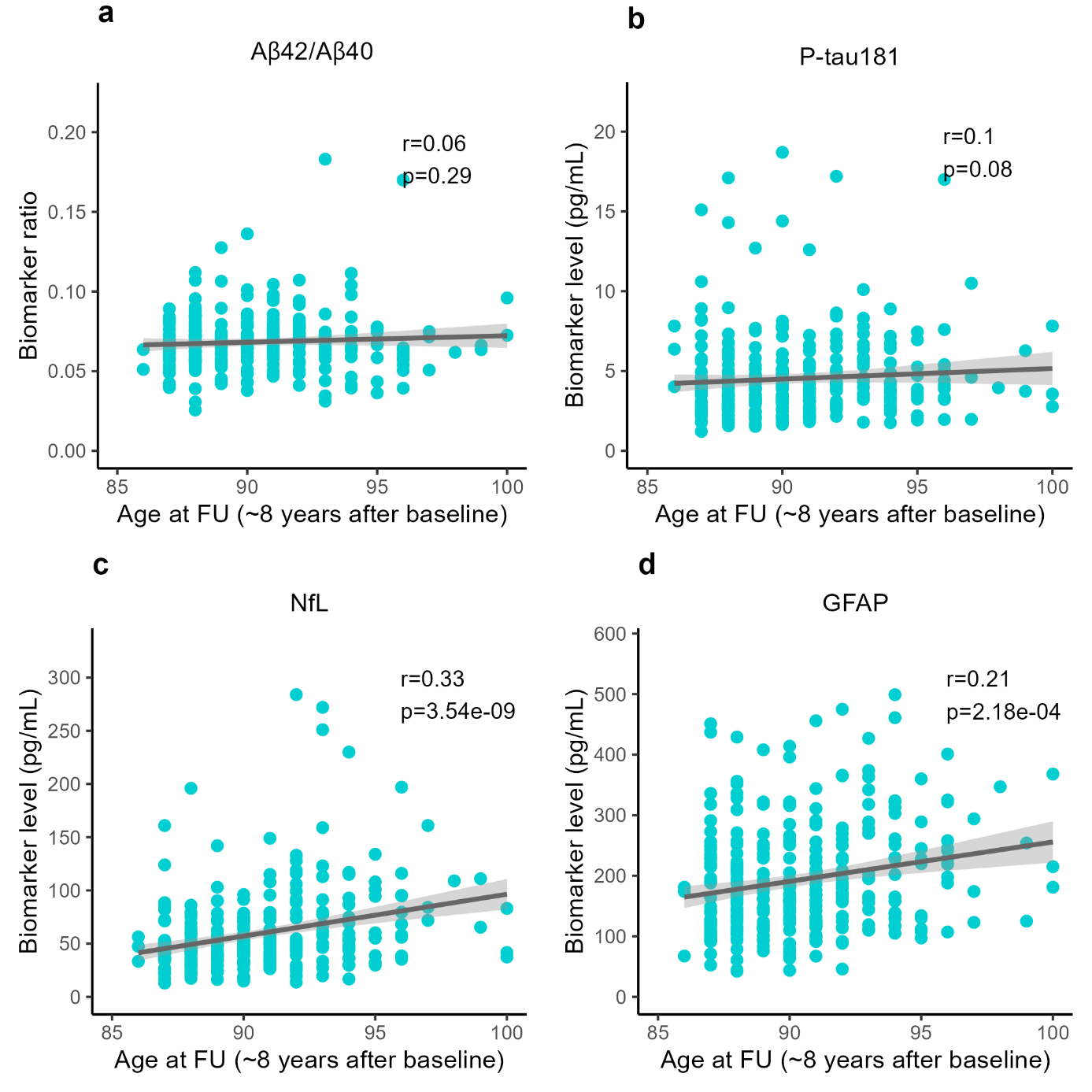
**

**Supplementary Figure 4. Correlation of follow-up plasma biomarkers with age in the subset of participants without dementia with a follow-up sample.** Scatter plots show the association between age at follow-up and follow-up plasma Aβ42/Aβ40 ratio (a), or plasma levels of P-tau181 (b), NfL (c), and GFAP (d). FU, follow-up; r, Pearson correlation coefficient.

**Supplementary Table 1. Association between plasma biomarker levels and cognitive decline in participants without dementia.**

|  | **Aβ42/Aβ40**  **N=827** | | | **P-tau181**  **N=915** | | | **NfL**  **N=924** | | | **GFAP**  **N=925** | | |
| --- | --- | --- | --- | --- | --- | --- | --- | --- | --- | --- | --- | --- |
| **Variables** | **β** | **SE** | ***p*-value** | **β** | **SE** | ***p*-value** | **β** | **SE** | ***p*-value** | **β** | **SE** | ***p*-value** |
| Time (years) | -2∙27 | 0∙19 | 1∙83x10^-31^ | -2∙36 | 0∙18 | 1∙64x10^-36^ | -2∙25 | 0∙18 | 8∙67x10^-34^ | -2∙18 | 0∙17 | 6∙58x10^-33^ |
| Biomarker ratio/level | 1∙14 | 0∙42 | 6∙79x10^-3^ | 0∙43 | 0∙40 | 0∙28 | -0∙33 | 0∙40 | 0∙42 | -0∙31 | 0∙41 | 0∙94 |
| Age (baseline) | -2∙33 | 0∙42 | 5∙40x10^-8^ | -2∙46 | 0∙40 | 1∙03x10^-9^ | -0∙24 | 0∙40 | 5∙81x10^-9^ | -2∙39 | 0∙4 | 3∙94x10^-9^ |
| Sex (male) | -2∙16 | 0∙91 | 0∙02 | -2∙22 | 0∙88 | 0∙01 | -0∙22 | 0∙86 | 0∙01 | -2∙11 | 0∙87 | 0∙02 |
| *APOE*-ε2 | -1∙19 | 1∙13 | 0∙29 | -1∙05 | 1∙08 | 0∙33 | -0∙98 | 1∙07 | 0∙36 | -0∙94 | 1∙08 | 0∙39 |
| *APOE*-ε4 | -1∙49 | 1∙12 | 0∙18 | -1∙99 | 1∙07 | 0∙06 | -1∙79 | 1∙06 | 0∙09 | -1∙86 | 1∙05 | 0∙08 |
| Education level (intermediate) | 4∙04 | 0∙94 | 2∙02x10^-5^ | 3∙76 | 0∙89 | 2∙87x10^-5^ | 4∙05 | 0∙89 | 6∙11x10^-6^ | 3∙99 | 0∙89 | 8∙00 x10^-6^ |
| Education level (high) | 10∙27 | 1∙33 | 3∙34x10^-14^ | 9∙82 | 1∙25 | 1∙23x10^-14^ | 10∙17 | 1∙24 | 6∙40x10^-16^ | 10∙09 | 1∙24 | 1∙06x10^-15^ |
| Time x Biomarker ratio/level | 0∙10 | 0∙12 | 0∙40 | -0∙49 | 0∙12 | 2∙53x10^-5^ | -0∙29 | 0∙12 | 0∙01 | -0∙60 | 0∙12 | 1∙99x10^-7^ |
| Time x Age (baseline) | -0∙32 | 0∙12 | 7∙65x10^-3^ | -0∙25 | 0∙11 | 0∙03 | -0∙26 | 0∙11 | 0∙03 | -0∙19 | 0∙11 | 0∙09 |
| Time x Sex (male) | 0∙51 | 0∙25 | 0∙05 | 0∙78 | 0∙25 | 1∙65x10^-3^ | 0∙61 | 0∙24 | 0∙01 | 0∙38 | 0∙24 | 0∙11 |
| Time x *APOE*-ε2 | 0∙06 | 0∙31 | 0∙84 | 0∙04 | 0∙30 | 0∙88 | 1∙58x10^-3^ | 0∙30 | 1 | -0∙22 | 0∙3 | 0∙47 |
| Time x *APOE*-ε4 | -0∙61 | 0∙31 | 0∙05 | -0∙65 | 0∙30 | 0∙03 | -0∙69 | 0∙30 | 0∙02 | -0∙68 | 0∙29 | 0∙02 |
| Time x Education level (medium) | 0∙37 | 0∙26 | 0∙15 | 0∙21 | 0∙25 | 0∙39 | 0∙17 | 0∙25 | 0∙49 | 0∙21 | 0∙25 | 0∙39 |
| Time x Education level (high) | -0∙11 | 0∙36 | 0∙77 | -0∙36 | 0∙34 | 0∙29 | -0∙39 | 0∙34 | 0∙25 | -0∙28 | 0∙34 | 0∙41 |

Normalised MMSE score was used as the outcome measure. *APOE*-ε3 (ε3/ε3) was used as the reference *APOE* stratum. *APOE*-ε2 stratum included ε2/ε2 and ε2/ε3 genotypes, and *APOE*-ε4, ε2/ε4, ε3/ε4, ε4/ε4 genotypes. Education was categorised into elementary, intermediate, and high levels using the CASMIN international educational classification. Biomarker ratio/levels and age were *z*-transformed to allow comparison.

**Supplementary Table 2. Association between plasma biomarker levels and cognitive decline in participants without cognitive impairment.**

|  | **Aβ42/Aβ40**  **N=743** | | | **P-tau181**  **N=823** | | | **NfL**  **N=834** | | | **GFAP**  **N=836** | | |
| --- | --- | --- | --- | --- | --- | --- | --- | --- | --- | --- | --- | --- |
| **Variables** | **β** | **SE** | ***p*-value** | **β** | **SE** | ***p*-value** | **β** | **SE** | ***p*-value** | **β** | **SE** | ***p*-value** |
| Time (years) | -2∙17 | 0∙19 | 1∙48x10^-26^ | -2∙21 | 0∙18 | 3∙69x10^-30^ | -2∙11 | 0∙18 | 4∙49x10^-28^ | -2∙07 | 0∙18 | 1∙43x10^-27^ |
| Biomarker ratio/level | 0∙62 | 0∙41 | 0∙13 | 0∙44 | 0∙39 | 0∙26 | 0∙22 | 0∙39 | 0∙58 | 0∙41 | 0∙39 | 0∙3 |
| Age (baseline) | -2∙15 | 0∙41 | 2∙65x10^-7^ | -2∙29 | 0∙39 | 5∙03x10^-9^ | -2∙26 | 0∙39 | 9∙04x10^-9^ | -2∙31 | 0∙39 | 4∙67x10^-9^ |
| Sex (male) | -3∙30 | 0∙88 | 2∙05x10^-4^ | -3∙11 | 0∙85 | 2∙91x10^-4^ | -3∙16 | 0∙84 | 1∙69x10^-4^ | -2∙96 | 0∙84 | 4∙65x10^-4^ |
| *APOE*-ε2 | -0∙82 | 1∙11 | 0∙46 | -0∙45 | 1∙07 | 0∙67 | -0∙37 | 1∙06 | 0∙73 | -0∙31 | 1∙07 | 0∙77 |
| *APOE*-ε4 | -1∙99 | 1∙08 | 0∙07 | -2∙32 | 1∙03 | 0∙02 | -2∙19 | 1∙02 | 0∙03 | -2∙22 | 1∙02 | 0∙03 |
| Education level (intermediate) | 5∙31 | 0∙94 | 2∙69x10^-8^ | 4∙91 | 0∙9 | 5∙40x10^-8^ | 5∙09 | 0∙89 | 1∙63x10^-8^ | 4∙99 | 0∙89 | 2∙83x10^-8^ |
| Education level (high) | 10∙04 | 1∙27 | 9∙35x10^-15^ | 9∙55 | 1∙2 | 6∙29x10^-15^ | 9∙90 | 1∙19 | 2∙92x10^-16^ | 9∙77 | 1∙19 | 6∙58x10^-16^ |
| Time x Biomarker ratio/level | 0∙11 | 0∙12 | 0∙35 | -0∙42 | 0∙12 | 5∙45x10^-4^ | -0∙26 | 0∙12 | 0∙03 | -0∙52 | 0∙12 | 1∙38x10^-5^ |
| Time x Age (baseline) | -0∙34 | 0∙13 | 6∙83x10^-3^ | -0∙27 | 0∙12 | 0∙02 | -0∙28 | 0∙12 | 0∙02 | -0∙23 | 0∙12 | 0∙05 |
| Time x Sex (male) | 0∙35 | 0∙27 | 0∙20 | 0∙59 | 0∙26 | 0∙02 | 0∙45 | 0∙25 | 0∙08 | 0∙28 | 0∙25 | 0∙26 |
| Time x APOE2 | -0∙21 | 0∙34 | 0∙52 | -0∙24 | 0∙32 | 0∙45 | -0∙27 | 0∙32 | 0∙40 | -0∙41 | 0∙32 | 0∙20 |
| Time x APOE4 | -0∙70 | 0∙33 | 0∙03 | -0∙77 | 0∙31 | 0∙01 | -0∙80 | 0∙31 | 9∙67x10^-3^ | -0∙79 | 0∙31 | 9∙93x10^-3^ |
| Time x Education level (medium) | 0∙17 | 0∙28 | 0∙55 | -0∙03 | 0∙27 | 0∙92 | -0∙06 | 0∙27 | 0∙82 | -1∙89x10^-4^ | 0∙27 | 1 |
| Time x Education level (high) | -0∙16 | 0∙38 | 0∙67 | -0∙41 | 0∙36 | 0∙25 | -0∙44 | 0∙35 | 0∙21 | -0∙36 | 0∙35 | 0∙30 |

Normalised MMSE score was used as the outcome measure. *APOE*-ε3 (ε3/ε3) was used as the reference *APOE* stratum. *APOE*-ε2 stratum included ε2/ε2 and ε2/ε3 genotypes, and *APOE*-ε4, ε2/ε4, ε3/ε4, ε4/ε4 genotypes. Education was categorised into elementary, intermediate, and high levels using the CASMIN international educational classification. Biomarker ratio/levels and age were *z*-transformed to allow comparison.

**Supplementary Table 3. Association between baseline plasma biomarker ratio or levels and risk of progressing to clinically-diagnosed dementia of the Alzheimer’s type in participants without cognitive impairment.**

|  | **Aβ42/Aβ40**  **N=769** | | | **P-tau181**  **N=855** | | | **NfL**  **N=862** | | | **GFAP**  **N=864** | | |
| --- | --- | --- | --- | --- | --- | --- | --- | --- | --- | --- | --- | --- |
| **Variables** | **HR** | **95% CI** | ***p*-value** | **HR** | **95% CI** | ***p*-value** | **HR** | **95% CI** | ***p*-value** | **HR** | **95% CI** | ***p*-value** |
| Biomarker | 0∙78 | 0∙65-0∙94 | 0∙01 | 1∙34 | 1∙17-1∙54 | 3∙21x10^-5^ | 1∙14 | 0∙98-1∙34 | 0∙09 | 1∙32 | 1∙16-1∙51 | 2∙09x10^-5^ |
| Age | 1∙51 | 1∙29-1∙77 | 2∙47x10^-7^ | 1∙49 | 1∙29-1∙71 | 6∙08x10^-8^ | 1∙47 | 1∙27-1∙7 | 1∙59x10^-7^ | 1∙41 | 1∙22-1∙64 | 2∙90x10^-6^ |
| Sex (male) | 0∙73 | 0∙49-1∙10 | 0∙13 | 0∙58 | 0∙39-0∙87 | 7∙38x10^-3^ | 0∙70 | 0∙48-1∙02 | 0∙06 | 0∙76 | 0∙52-1∙11 | 0∙15 |
| eGFR | 1∙02 | 0∙85-1∙22 | 0∙84 | 1∙17 | 0∙99-1∙39 | 0∙07 | 1∙11 | 0∙93-1∙32 | 0∙26 | 1∙09 | 0∙93-1∙29 | 0∙28 |

eGFR, estimated glomerular filtration rate; HR, hazard ratio; CI, confidence interval. All continuous variables were *z*-transformed to allow comparison.

**Supplementary Table 4. Association between baseline plasma biomarker ratio or levels and risk of progressing to mild cognitive impairment.**

|  | **Aβ42/Aβ40**  **N=755** | | | **P-tau181**  **N=840** | | | **NfL**  **N=846** | | | **GFAP**  **N=848** | | |
| --- | --- | --- | --- | --- | --- | --- | --- | --- | --- | --- | --- | --- |
| **Variables** | **HR** | **95% CI** | ***p*-value** | **HR** | **95% CI** | ***p*-value** | **HR** | **95% CI** | ***p*-value** | **HR** | **95% CI** | ***p*-value** |
| Biomarker | 0∙90 | 0∙80-1∙02 | 0∙11 | 1∙16 | 1∙04-1∙29 | 8∙03x10^-3^ | 1∙04 | 0∙92-1∙17 | 0∙54 | 1∙20 | 1∙08-1∙33 | 4∙43x10^-4^ |
| Age | 1∙45 | 1∙28-1∙6 | 2∙11x10^-9^ | 1∙44 | 1∙29-1∙61 | 5∙91x10^-11^ | 1∙44 | 1∙29-1∙61 | 1∙21x10^-10^ | 1∙42 | 1∙27-1∙59 | 5∙21x10^-10^ |
| Sex (male) | 1∙13 | 0∙87-1∙46 | 0∙35 | 1∙02 | 0∙79-1∙31 | 0∙89 | 1∙12 | 0∙88-1∙42 | 0∙37 | 1∙15 | 0∙91-1∙47 | 0∙25 |
| eGFR | 1∙02 | 0∙90-1∙16 | 0∙74 | 1∙07 | 0∙95-1∙20 | 0∙28 | 1∙03 | 0∙91-1∙17 | 0∙66 | 1∙05 | 0∙93-1∙18 | 0∙41 |

eGFR, estimated glomerular filtration rate; HR, hazard ratio; CI, confidence interval. All continuous variables were *z*-transformed to allow comparison.

**Supplementary Table 5. Association of different combinations of cognitive testing and plasma biomarkers with probability of developing clinically-diagnosed dementia of the Alzheimer's type.**

|  | **All participants**  **(N=809)** | | | | | | | | | **Participants with MMSE≤27**  **(N=192)** | | | | | | | | |
| --- | --- | --- | --- | --- | --- | --- | --- | --- | --- | --- | --- | --- | --- | --- | --- | --- | --- | --- |
|  | **MMSE** | | | **Biomarkers** | | | **MMSE + biomarkers** | | | **MMSE** | | | **Biomarkers** | | | **MMSE + biomarkers** | | |
| **Variables** | **β** | **SE** | ***p*-value** | **β** | **SE** | ***p*-value** | **β** | **SE** | ***p*-value** | **β** | **SE** | ***p*-value** | **β** | **SE** | ***p*-value** | **β** | **SE** | ***p*-value** |
| Age | 0∙32 | 0∙11 | 0∙01 | 0∙34 | 0∙11 | 1∙95x10^-3^ | 0∙26 | 0∙11 | 0∙02 | 0∙05 | 0∙18 | 0∙79 | 0∙12 | 0∙19 | 0∙53 | 0∙03 | 0∙19 | 0∙90 |
| Sex (male) | -0∙64 | 0∙28 | 0∙02 | -0∙67 | 0∙30 | 0∙02 | -0∙64 | 0∙30 | 0∙03 | -1∙02 | 0∙46 | 0∙03 | -1∙11 | 0∙48 | 0∙03 | -1∙08 | 0∙49 | 0∙03 |
| *APOE*-ε2 | -0∙28 | 0∙35 | 0∙42 | -0∙08 | 0∙35 | 0∙83 | -0∙19 | 0∙36 | 0∙60 | -1∙48 | 0∙67 | 0∙03 | -1∙44 | 0∙68 | 0∙03 | -1∙69 | 0∙73 | 0∙02 |
| *APOE*-ε4 | 0∙66 | 0∙28 | 0∙02 | 0∙67 | 0∙28 | 0∙02 | 0∙62 | 0∙28 | 0∙03 | 0∙11 | 0∙43 | 0∙79 | -0∙17 | 0∙44 | 0∙70 | -0∙05 | 0∙45 | 0∙92 |
| Aβ42/Aβ40 | - | - | - | -0∙29 | 0∙13 | 0∙03 | -0∙22 | 0∙13 | 0∙08 | - | - | - | -0∙51 | 0∙21 | 0∙02 | -0∙49 | 0∙22 | 0∙02 |
| P-tau181 | - | - | - | 0∙14 | 0∙12 | 0∙26 | 0∙16 | 0∙12 | 0∙18 | - | - | - | 0∙25 | 0∙19 | 0∙20 | 0∙28 | 0∙19 | 0∙13 |
| NfL | - | - | - | -0∙07 | 0∙13 | 0∙63 | -0∙09 | 0∙13 | 0∙49 | - | - | - | 0∙08 | 0∙18 | 0∙66 | 0∙06 | 0∙18 | 0∙75 |
| GFAP | - | - | - | 0∙27 | 0∙11 | 0∙01 | 0∙27 | 0∙11 | 0∙02 | - | - | - | 0∙13 | 0∙19 | 0∙48 | 0∙15 | 0∙19 | 0∙43 |
| MMSE (normalised) | -0∙69 | 0∙12 | 2∙79x10^-8^ | - | - | - | -0∙66 | 0∙12 | 1∙13x10^-7^ | -0∙43 | 0∙17 | 0∙01 | - | - | - | -0∙44 | 0∙18 | 0∙01 |

*APOE*-ε3 (ε3/ε3) was used as the reference *APOE* stratum. *APOE*-ε2 stratum included ε2/ε2 and ε2/ε3 genotypes, and *APOE*-ε4, ε2/ε4, ε3/ε4, ε4/ε4 genotypes. MMSE score was normalised to avoid ceiling effects. All continuous variables were *z*-transformed to allow comparison.

**Supplementary Table 6. Performance of different simplified strategies to predict future clinically-diagnosed dementia of Alzheimer’s type.**

|  | **Model parameters** | | | | | | | | |
| --- | --- | --- | --- | --- | --- | --- | --- | --- | --- |
| **Strategy** | **TP** | **FP** | **TN** | **FN** | **PPV** | **NPV** | **Sens** | **Spec** | **AUC** |
| All participants (N=809; DAT-progressors, N=89) |  |  |  |  |  |  |  |  |  |
| Aβ42/Aβ40 + GFAP + age + sex + *APOE* strata | 64 | 248 | 472 | 25 | 0∙21 | 0∙95 | 0∙72 | 0∙66 | 0∙72 |
| MMSE + Aβ42/Aβ40 + GFAP + age + sex + *APOE* strata | 76 | 301 | 419 | 13 | 0∙21 | 0∙97 | 0∙85 | 0∙58 | 0∙77 |
| Aβ42/Aβ40 + GFAP | 44 | 151 | 569 | 45 | 0∙23 | 0∙93 | 0∙49 | 0∙79 | 0∙67 |
| MMSE + Aβ42/Aβ40 + GFAP | 45 | 97 | 623 | 44 | 0∙32 | 0∙93 | 0∙51 | 0∙87 | 0∙73 |
| Participants with MMSE≤27 (N=192; DAT-progressors, N=45) |  |  |  |  |  |  |  |  |  |
| Aβ42/Aβ40 + GFAP + age + sex + *APOE* strata | 35 | 59 | 88 | 10 | 0∙37 | 0∙90 | 0∙78 | 0∙60 | 0∙73 |
| MMSE + Aβ42/Aβ40 + GFAP + age + sex + *APOE* strata | 27 | 27 | 120 | 18 | 0∙50 | 0∙87 | 0∙60 | 0∙82 | 0∙74 |
| Aβ42/Aβ40 + GFAP | 38 | 77 | 70 | 7 | 0∙33 | 0∙91 | 0∙84 | 0∙48 | 0∙68 |
| MMSE + Aβ42/Aβ40 + GFAP | 34 | 51 | 96 | 11 | 0∙40 | 0∙90 | 0∙76 | 0∙65 | 0∙71 |

DAT, clinically-diagnosed dementia of the Alzheimer’s type; TP, true positive cases; FP, false positive cases; TN, true negative cases; FN, false negative cases; PPV, positive predictive value; NPV, negative predictive value; Sens, sensitivity; Spec, specificity; AUC, area under the ROC curve.

**Supplementary Table 7. Association of different simplified combinations of cognitive testing and plasma biomarkers with probability of developing clinically-diagnosed dementia of the Alzheimer's type.**

|  | **Predictive models** | | | | | | | | | | | |
| --- | --- | --- | --- | --- | --- | --- | --- | --- | --- | --- | --- | --- |
|  | **Aβ42/Aβ40 + GFAP +**  **age + sex + APOE strata** | | | **MMSE + Aβ42/Aβ40 + GFAP +**  **age + sex + APOE strata** | | | **Aβ42/Aβ40 + GFAP** | | | **MMSE + Aβ42/Aβ40 + GFAP** | | |
| **Variables** | **β** | **SE** | ***p*-value** | **β** | **SE** | ***p*-value** | **β** | **SE** | ***p*-value** | **β** | **SE** | ***p*-value** |
| All participants (N=809; DAT progression, N=89) |  |  |  |  |  |  |  |  |  |  |  |  |
| Age | 0∙34 | 0∙11 | 1∙95x10^-3^ | 0∙26 | 0∙11 | 0∙02 | - | - | - | - | - | - |
| Sex (male) | -0∙59 | 0∙28 | 0∙04 | -0∙54 | 0∙29 | 0∙06 | - | - | - | - | - | - |
| *APOE*-ε2 | -0∙07 | 0∙34 | 0∙84 | -0∙18 | 0∙35 | 0∙61 | - | - | - | - | - | - |
| *APOE*-ε4 | 0∙68 | 0∙27 | 0∙01 | 0∙64 | 0∙28 | 0∙02 | - | - | - | - | - | - |
| Aβ42/Aβ40 | -0∙30 | 0∙13 | 0∙02 | -0∙23 | 0∙13 | 0∙08 | -0∙32 | 0∙13 | 0∙01 | -0∙23 | 0∙13 | 0∙08 |
| GFAP | 0∙30 | 0∙10 | 2∙28x10^-3^ | 0∙29 | 0∙10 | 4∙80x10^-3^ | 0∙39 | 0∙09 | 2∙16x10^-5^ | 0∙37 | 0∙10 | 9∙69x10^-5^ |
| MMSE (normalised) | - | - | - | -0∙65 | 0∙12 | 1∙45x10^-7^ | - | - | - | -0∙69 | 0∙12 | 1∙17x10^-8^ |
| Participants with MMSE≤27 (N=192; DAT progression, N=45) |  |  |  |  |  |  |  |  |  |  |  |  |
| Age | 0∙11 | 0∙19 | 0∙57 | 0∙02 | 0∙19 | 0∙93 | - | - | - | - | - | - |
| Sex (male) | -1∙01 | 0∙47 | 0∙03 | -0∙96 | 0∙48 | 0∙04 | - | - | - | - | - | - |
| *APOE*-ε2 | -1∙33 | 0∙66 | 0∙04 | -1∙49 | 0∙68 | 0∙03 | - | - | - | - | - | - |
| *APOE*-ε4 | -0∙08 | 0∙44 | 0∙86 | 0∙06 | 0∙44 | 0∙90 | - | - | - | - | - | - |
| Aβ42/Aβ40 | -0∙49 | 0∙21 | 0∙02 | -0∙46 | 0∙21 | 0∙03 | -0∙38 | 0∙20 | 0∙05 | -0∙38 | 0∙20 | 0∙06 |
| GFAP | 0∙22 | 0∙18 | 0∙23 | 0∙23 | 0∙18 | 0∙19 | 0∙39 | 0∙17 | 0∙02 | 0∙38 | 0∙17 | 0∙02 |
| MMSE (normalised) | - | - | - | -0∙42 | 0∙18 | 0∙02 | - | - | - | -0∙36 | 0∙17 | 0∙03 |

*APOE*-ε3 (ε3/ε3) was used as the reference *APOE* stratum. *APOE*-ε2 stratum included ε2/ε2 and ε2/ε3 genotypes, and *APOE*-ε4, ε2/ε4, ε3/ε4, ε4/ε4 genotypes. MMSE score was normalised to avoid ceiling effects. All continuous variables were *z*-transformed to allow comparison.

**Supplementary Table 8. Dynamics of plasma biomarkers in a subset of participants without dementia over the observational period.**

|  | **Aβ42/Aβ40**  **N=283** | | | **P-tau181**  **N=284** | | | **NfL**  **N=287** | | | **GFAP**  **N=287** | | |
| --- | --- | --- | --- | --- | --- | --- | --- | --- | --- | --- | --- | --- |
| **Variables** | **β** | **SE** | ***p*-val** | **β** | **SE** | ***p*-val** | **β** | **SE** | ***p*-val** | **β** | **SE** | ***p*-val** |
| Time (years) | -6∙89x10^-4^ | 2∙15x10^-4^ | 1∙54x10^-3^ | 0∙19 | 0∙03 | 1∙83x10^-11^ | 2∙88 | 0∙36 | 2∙44x10^-14^ | 8∙23 | 0∙77 | 1∙60x10^-22^ |
| Age (baseline) | 2∙34x10^-4^ | 1∙07x10^-3^ | 0∙83 | -4∙60x10^-3^ | 0∙13 | 0∙97 | 1∙57 | 1∙64 | 0∙34 | 7∙87 | 4∙16 | 0∙06 |
| Sex (male) | -2∙59x10^-3^ | 2∙18x10^-3^ | 0∙24 | 0∙59 | 0∙26 | 0∙02 | 5∙27 | 3∙38 | 0∙12 | -11∙24 | 8∙55 | 0∙19 |
| *APOE-ε*2 | -8∙44x10^-5^ | 2∙85x10^-3^ | 0∙98 | -0∙07 | 0∙34 | 0∙83 | -4∙18 | 4∙38 | 0∙34 | -18∙30 | 11∙15 | 0∙1 |
| *APOE-ε4* | -5∙60x10^-3^ | 2∙88x10^-3^ | 0∙05 | 0∙1 | 0∙34 | 0∙78 | -1∙83 | 4∙44 | 0∙68 | -6∙49 | 11∙24 | 0∙56 |
| eGFR (mL/min/BSA) | 6∙53x10^-4^ | 1∙09x10^-3^ | 0∙55 | -0∙57 | 0∙13 | 1∙23x10^-5^ | -8∙98 | 1∙66 | 8∙87x10^-8^ | -13∙98 | 4∙19 | 9∙22x10^-4^ |
| Progression (DAT) | -9∙57x10^-3^ | 3∙11x10^-3^ | 2∙21x10^-3^ | 0∙47 | 0∙36 | 0∙19 | 8∙23 | 4∙65 | 0∙08 | 37∙25 | 11∙77 | 1∙66x10^-3^ |
| Time x Age (baseline) | -1∙19x10^-5^ | 1∙56x10^-4^ | 0∙94 | 0∙02 | 0∙02 | 0∙20 | 0∙96 | 0∙25 | 1∙61x10^-4^ | 0∙94 | 0∙55 | 0∙09 |
| Time x Sex (male) | 1∙41x10^-4^ | 3∙13x10^-4^ | 0∙65 | 0∙06 | 0∙04 | 0∙13 | -0∙40 | 0∙52 | 0∙44 | -2∙30 | 1∙11 | 0∙04 |
| Time x *APOE-ε*2 | 4∙47x10^-4^ | 4∙12x10^-4^ | 0∙28 | -0∙07 | 0∙05 | 0∙16 | -0∙54 | 0∙67 | 0∙43 | 1∙66 | 1∙46 | 0∙26 |
| Time x *APOE-ε*4 | -8∙65x10^-5^ | 4∙17x10^-4^ | 0∙84 | 0∙02 | 0∙05 | 0∙73 | 0∙25 | 0∙69 | 0∙72 | 2∙10 | 1∙48 | 0∙16 |
| Time x eGFR | 3∙85x10^-6^ | 1∙54x10^-4^ | 0∙98 | -7∙44x10^-3^ | 0∙02 | 0∙70 | -0∙18 | 0∙25 | 0∙48 | 0∙54 | 0∙54 | 0∙32 |
| Time x progression (DAT) | 6∙18x10^-4^ | 4∙64x10^-4^ | 0∙18 | 9∙74x10^-3^ | 0∙06 | 0∙86 | 2∙47 | 0∙72 | 7∙38x10^-4^ | 4∙45 | 1∙56 | 4∙59x10^-3^ |

Longitudinal plasma ratio or levels were used as the outcome measure. All continuous independent variables were *z*-transformed to allow comparison. eGFR, estimated glomerular filtration rate; BSA, body surface area (1∙73 m^2^); DAT, clinically-diagnosed dementia of the Alzheimer’s type.

**References**

1 Jessen F, Wiese B, Cvetanovska G, *et al.* Patterns of subjective memory impairment in the elderly: Association with memory performance. *Psychol Med* 2007; **37**: 1753–62.

2 Luck T, Riedel-Heller SG, Kaduszkiewicz H, *et al.* Mild cognitive impairment in general practice: Age-specific prevalence and correlate results from the German study on ageing, cognition and dementia in primary care patients (AgeCoDe). *Dement Geriatr Cogn Disord* 2007; **24**: 307–16.

3 Zaudig M, Mittelhammer J, Hiller W, *et al.* SIDAM — A Structured Interview for the diagnosis of Dementia of the Alzheimer type, Multi-infarct dementia and dementias of other aetiology according to ICD-10 and DSM-III-R. *Psychol Med* 1991. DOI:10.1017/S0033291700014811.

4 Morris JC, Heyman A, Mohs RC, *et al.* The consortium to establish a registry for alzheimer’s disease (CERAD). Part I. Clinical and neuropsychological assessment of alzheimer’s disease. *Neurology* 1989. DOI:10.1212/wnl.39.9.1159.

5 Reisberg B, Ferris SH, de Leon MJ, Crook T. The Global Deterioration Scale for assessment of primary degenerative dementia. *Am J Psychiatry* 1982; **139**: 1136–9.

6 Blessed G, Tomlinson BE, Roth M. The association between quantitative measures of dementia and of senile change in the cerebral grey matter of elderly subjects. *Br J Psychiatry* 1968. DOI:10.1192/bjp.114.512.797.

7 Brauns H, Steinmann S. Educational reform in France, West-Germany and the United Kingdom: updating the CASMIN educational classification. *ZUMA Nachrichten* 1999; **23**: 7–44.
